# Transforming cisplatin into targeted photothermal chemotherapeutics through the platinum-phosphate coordination within a hyaluronan nanogel

**DOI:** 10.1126/sciadv.adz7615

**Published:** 2026-03-18

**Authors:** Yiyi Zhang, Huimin Wang, Hua Guo, Xiaorong Gou, Xinxin Hao, Jiayi Li, Hong Deng, Weiqi Zhang

**Affiliations:** ^1^ State Key Laboratory of Complex Severe and Rare Diseases, Institute of Basic Medical Sciences, Chinese Academy of Medical Sciences and Peking Union Medical College, Beijing, 100005, P. R. China.; ^2^ State Key Laboratory of Molecular Oncology and Department of Molecular Oncology, National Cancer Center/National Clinical Research Center for Cancer/Cancer Hospital, Chinese Academy of Medical Sciences and Peking Union Medical College, Beijing 100021, P. R. China.

## Abstract

Cisplatin’s (Cis’s) antitumor efficacy is limited by dose-dependent toxicity due to nonspecific tissue distribution. While targeted Cis delivery and combination therapies boost therapeutic efficacy, developing multifunctional yet simplified formulations remains challenging. Inspired by the Cis’s interaction with DNA, here, we transformed Cis into a reversible photothermal agent through the platinum-phosphate (Pt-phos) interaction within a hyaluronan (HA) nanogel (named as HA/PtP) to afford synergistic photothermal therapy (PTT) and chemotherapy. The HA/PtP nanogel exhibits near infrared absorption and 32.08% photothermal conversion efficiency, and degrades to release Cis through the reversible Pt-phos interaction under acidic conditions. Leveraging HA’s intrinsic CD44 targeting, HA/PtP nanogel simultaneously enables highly efficient PTT and targeted chemotherapy in 4T1 breast tumor xenografts. Moreover, HA/PtP-based PTT successfully mimics hyperthermic intraperitoneal chemotherapy (HIPEC) but with alleviated Cis toxicity in MC38 metastatic mouse model. This HA/PtP nanogel represents a rationally designed Cis nanomedicine integrating the PTT with self-targeting and facile preparation.

## Teaser

The platinum-phosphate coordination within HA nanogel converts cisplatin into a targeted anticancer photothermal chemotherapeutic.

## INTRODUCTION

Cisplatin (Cis), a platinum (Pt) coordination complex, was first demonstrated to have a potent anticancer effect and then approved by the US Food and Drug Administration in 1978 (*1*, *2*). However, the systemic toxicities associated with Cis treatment can lead to severe adverse effects in cancer patients, which notably limit its clinical efficacy (*3*–*5*). Currently, there is a significant focus on developing new approaches to reduce the systemic side effects of Cis and enhance the potency of cancer therapies. Targeted delivery represents a highly active area of research in anticancer drug development, demonstrating two major advantages: selective delivery to tumor cells and reduced damage to normal tissues (*6*, *7*). Various targeted nanocarriers have been developed to load Cis either through its coordination with carriers or via direct encapsulation. Typical examples include liposomes (*8*, *9*), micelles (*7*, *10*), and nanogels (*11*–*13*), to name a few. Moreover, a Cis delivery that could be activated under tumor microenvironments (TMEs), such as the acidic pH-responsive delivery, could further improve the selective chemotoxicity and reduce the systemic side effects (*7*, *14*).

Besides the efforts to improve the Cis delivery, a combination of Cis chemotherapy with other therapeutic modalities has also been widely exploited to improve the anticancer outcome. One typical benefit is that adding an additional treatment modality, such as phototherapy, can facilitate tumor ablation through light-directed cell killing, potentially allowing dose reduction of Cis and minimizing side effects. Photothermal therapy (PTT) has attracted tremendous attention as it converts optical energy into thermal energy using photothermal conversion agents (PTAs) under near-infrared (NIR) laser irradiation for tumor hyperthermia ablation (*15*–*17*). However, a common issue with PTT is the inability to induce complete tumor eradication due to the nonuniform heat distribution and heat endurance. Combining PTT with chemotherapy has been suggested as a viable strategy to resolve this issue, due to their complementary and synergistic effects (*18*, *19*). Regarding the combination of Cis chemotherapy and PTT, the classic strategy integrates Cis or its prodrug with a PTA, for example, MnO_2_ nanosheets (*20*), gold nanorods (*21*), etc. Despite the antitumor benefits of targeted multifunctional nanocarriers to combine PTT and Cis chemotherapy, rational designs are required to balance the therapeutic efficacy of Cis, the efficiency of PTT, and the potential nonspecific toxicity.

As a chemotherapeutic agent, Cis exerts its cytotoxicity primarily through binding to DNA (*2*, *22*). It mainly binds the N7 position of guanines and adenines in the major groove of the double helix, leading to the formation of cytotoxic cisplatin-DNA (Cis-DNA) adducts (*1*, *23*). Notably, Cis could also noncovalently interact with the phosphate group (Pt-phos interaction) of the DNA backbone through both coordination and hydrogen bonding (*24*, *25*). Similar to this process, Pt-phos interaction has been exploited to synthesize platinum-pyrophosphate prodrug (PT-112) (*26*), construct the self-assembled nanomedicine using Cis and bone-targeted phytic acid (*27*), and load Cis into black phosphorus nanosheets, which acted as PTA (*28*). In addition, the Pt-phos interaction is weakened under acidic conditions (*27*), a key feature within the TMEs and lysosomes in cells. While the above formulation strategy clearly facilitates the Cis treatment, it also highlights the great potential of leveraging the Pt-phos interaction to directly construct nanocarriers with inherent capabilities for both cancer-targeting and synergistic PTT/chemotherapy.

Hyaluronan (HA), a natural polysaccharide from the extracellular matrix of mammalian cells, can specifically target CD44, which is overexpressed on the surface of different cancer cells. Moreover, HA has a high density of carboxyl groups available for Cis coordination, which simultaneously allows Cis loading and the nanogel construction. Previously, we have reported a series of Cis–cross-linked HA nanogels to afford CD44-targeted therapeutic applications in different tumors (*12*, *29*–*31*). Here, inspired by the Pt-phos interaction involved in Cis’s DNA binding, we proposed a tumor-targeted HA/PtP nanogel for synergistic PTT/chemotherapy, which was synthesized by the self-assembly of HA, Cis, and phosphate group [provided by phosphate buffer (PB)]. The confinement of Pt-phos within the nanogel endows the HA/PtP with strong absorption in the NIR region and outstanding photothermal performance. The tumor-targeting ability of HA allowed HA/PtP nanogels to efficiently accumulate at the tumor site. Meanwhile, Pt-phos is reversible under acidic pH, leading to nanogel decomposition and Cis release within the TMEs, and subsequently enabling pH-responsive chemotherapy. This study demonstrates the direct conversion of the chemotherapeutic drug Cis into a functional PTA while retaining its intrinsic antitumor efficacy. The synergistic PTT/chemotherapy efficacy of HA/PtP was thus verified in a murine breast cancer xenograft model. Furthermore, its tumor-inhibitory performance was compared with that of hyperthermic intraperitoneal chemotherapy (HIPEC) (*32*, *33*)—a clinically approved regimen that relies on the intraperitoneal infusion of heated Cis—using an MC38 colorectal peritoneal metastasis model. Given the targeted delivery capability and pH-responsive characteristics of the HA/PtP system, its toxicity-reducing effect was systematically assessed against free Cis, with a focus on nephrotoxicity, as the kidneys are the primary organs affected by Cis-induced toxicity.

## RESULTS AND DISCUSSION

### Synthesis and characterization of HA/PtP nanogels

**Fig. 1.**
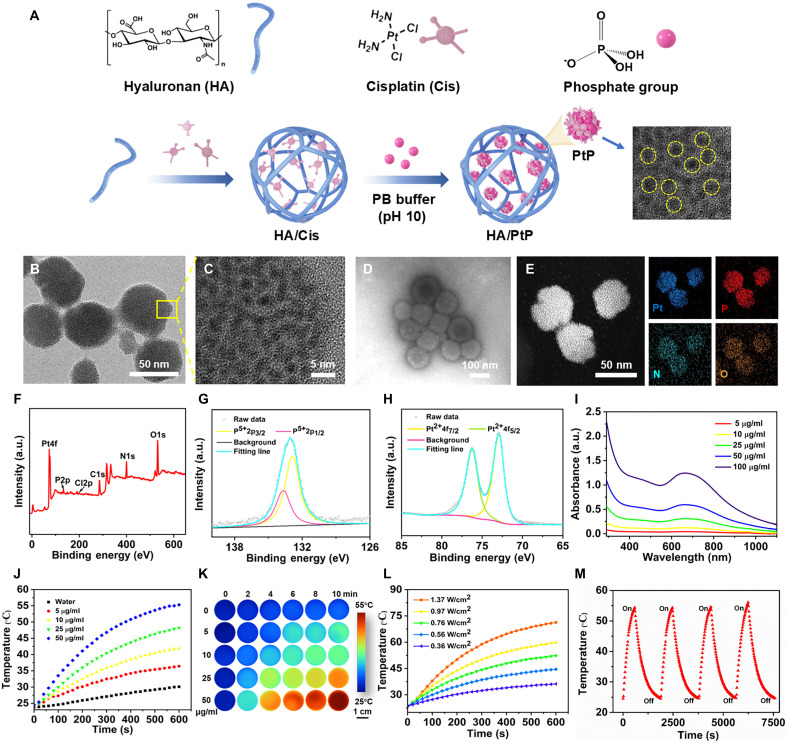
Preparation, characterization, and photothermal performance of HA/PtP nanogels. (**A**) Schematic illustration of the HA/PtP preparation. HA/Cis nanogel was prepared without introduction of PB. (**B**) TEM images of HA/PtP nanogels. (**C**) HRTEM images of HA/PtP nanogels. (**D**) TEM images of HA/PtP nanogels by negative staining with uranyl acetate. (**E**) STEM images and element mapping of HA/PtP nanogels. Blue, Pt; red, P; aqua, N; claybank, O. (**F**) XPS spectra of HA/PtP nanogels. High-resolution (**G**) P 2p and (**H**) Pt 4f XPS spectra of HA/PtP nanogels. (**I**) The UV-vis absorption spectra of HA/PtP nanogels with various concentrations (Pt element, 5, 10, 25, 50, and 100 μg/ml). (**J** and **K**) The photothermal heating curves and corresponding thermal images of HA/PtP solutions at different concentrations (Pt element, 0, 5, 10, 25, and 50 μg/ml) irradiated by an 808-nm laser (0.8 W/cm^2^). (**L**) Photothermal heating curves of an HA/PtP solution (Pt element, 50 μg/ml) under an 808-nm laser irradiation at different power densities (0.36, 0.56, 0.76, 0.97, and 1.37 W/cm^2^). (**M**) Photostability of HA/PtP solutions (Pt element, 50 μg/ml) under an 808-nm laser irradiation at 0.8 W/cm^2^. a.u., arbitrary unit.

The HA/PtP nanogel was simply prepared by heating the mixture of HA and Cis, followed by coincubation with PB (0.2 M, pH 10) at 90°C (Fig. 1A). While the assembly between HA and Cis generated a negatively charged HA/Cis nanogel with a hydrodynamic diameter of 54.6 ± 16.4 nm and a polydispersity index (PDI) of 0.795 ± 0.222 (table S1), further coincubation with PB not only increased the size to 205.3 ± 1.8 nm with a narrower size distribution (PDI = 0.046 ± 0.006) but also turned the solution color into dark black. In phosphate-buffered saline (PBS), the hydrodynamic size of HA/PtP was shrunk and then recovered when dispersed in deionized water (fig. S1). The repeated size elevation–reduction cycles of HA/PtP were maintained for at least four rounds following their transfer from PBS to water, indicating the nanogel feature of HA/PtP. The color change could be clearly reflected by the ultraviolet-visible (UV-vis) absorption spectra, as HA/Pt nanogel gained an NIR absorption peak (750 to 1100 nm) in reference to HA/Cis nanogel (fig. S2A), indicating the emergence of new photophysical properties possibly due to the Pt-phos interaction within the HA/PtP nanogel. As shown in Fig. 1B, transmission electron microscopy (TEM) images indicated that HA/PtP was successfully assembled, which appeared as spherical nanogels with a mean diameter of ~50 nm, and meanwhile, homogeneously distributed nanoclusters could be clearly spotted within the nanogel under the high-resolution TEM (HRTEM) (Fig. 1C). The TEM images of HA/PtP with negative staining showed that the diameter of nanogels increased to ~100 nm, which was likely attributed to the outer layer of HA stained by the uranyl acetate (Fig. 1D). The morphology of as-prepared HA/PtP nanogels could also be observed by scanning electron microscope (SEM) image (fig. S3A) and scanning transmission electron microscopy (STEM) image (Fig. 1E). Both the scanning electron microscopy with energy dispersive spectroscopy (SEM-EDS; fig. S3B) and the element mapping images (Fig. 1E) affirmed the colocalization of Pt and P that represents Cis and phosphate, suggesting that the Pt-phos were uniformly distributed in this nanogel. The x-ray photoelectron spectroscopy (XPS) analysis confirmed the successful assembly of HA/PtP nanogels (Fig. 1F). In the high-resolution XPS spectrum with the Cis and PB set as controls (figs. S4 and S5), the two strong binding energy peaks of HA/PtP at 72.98 eV (Pt^2+^4f_7/2_) and 76.18 eV (Pt^2+^4f_5/2_) corresponded to Pt^2+^of Cis (Fig. 1H and fig. S4), while the two prominent peaks of the P 2p at 132.88 eV (P^5+^2p_3/2_) and 133.68 eV (P^5+^2p_1/2_) were ascribed to P^5+^ of phosphate group (Fig. 1G and fig. S5); the spectra of C1s, N1s, and O1s were also analyzed (fig. S6). The above results indicated that there was no valence change of Pt during the assembly of HA/PtP nanogels. In Fourier transform infrared spectroscopy (FTIR) spectra of HA/Cis and HA/PtP, a blue shift from 1643 to 1620 cm^−1^ was observed, which could potentially be attributed to the creation of Pt-phos interaction (fig. S7) (*29*). The x-ray diffraction (XRD) was used to characterize the phase composition of HA/PtP nanogels (fig. S8). The thermogravimetry (TG) analysis with a heating rate of 15°C/min from room temperature to 1000°C in a N_2_ atmosphere was further demonstrated (fig. S9). The loss-on-ignition of HA/PtP nanogels was 66.54% in comparison with 82.15% of HA/Cis nanogels (table S2), which also indicated that the HA/PtP nanogel is more thermally stable due to the formed Pt-phos interaction within the nanogel.

### Photothermal performance of HA/PtP nanogels

As shown in Fig. 1I, the NIR absorption of HA/PtP nanogels further motivated us to investigate its photothermal performance. The HA/PtP nanogels aqueous solutions with different concentrations (Pt element, 0, 5, 10, 25, and 50 μg/ml) were recorded by exposing them to an 808-nm laser at a power density of 0.8 W/cm^2^. The temperature of all solutions with different concentrations of HA/PtP nanogels increased with time and showed a concentration-dependent growth pattern (Fig. 1, J and K). When the concentration of the HA/PtP nanogels reached 50 μg/ml (Pt element), the temperature of HA/PtP nanogels increased rapidly to 55.3°C from room temperature, while the temperature of water increased by only about 6.3°C. Further, the HA/PtP nanogels (Pt element, 50 μg/ml) aqueous solutions were irradiated with an 808-nm laser at different power densities (0.36, 0.56, 0.76, 0.97, and 1.37 W/cm^2^) (Fig. 1L), and it was found that the HA/PtP nanogels could effectively convert NIR light into heat energy, which could be accurately regulated. The laser on/off circulation curves clarified the superb photothermal stability and reproducibility of the HA/PtP nanogels, which remained a robust photothermal capability after four cycles under an 808-nm laser irradiation (Fig. 1M). The photothermal conversion efficiency (η) of the HA/PtP nanogels was calculated to be 32.08% on the basis of the photothermal heating curve and the cooling curve during laser on/off circulation (fig. S10) (*34*, *35*), which was obviously superior to those of typical PTAs such as Au nanorods (21%) (*36*), graphene quantum dots (28.58%) (*37*), and Ti_3_C_2_ nanosheets (30.6%) (*38*). According to the Lambert-Beer law, the extinction coefficient (ε) was determined to be 8.65 liter g^−1^ cm^−1^ at 808 nm (fig. S11). Moreover, density functional theory (DFT) calculations were carried out to elucidate the mechanism of the photothermal effect in HA/PtP. The calculated density of states reveals that the highest occupied molecular orbital–lowest unoccupied molecular orbital (HOMO-LUMO) gap of Cis is 6.07 eV, which decreases to 5.39 eV for HA/PtP upon the introduction of phosphate (fig. S12). The narrowed bandgap allowed NIR photogeneration of electrons and holes (*39*, *40*). Upon light absorption, the electrons transfer energy to surrounding atoms, converting light energy into thermal energy and thereby endowing HA/PtP with photothermal properties (*41*, *42*). Notably, the HA/PtP nanogels presented superb photothermal performance, when compared to HA/Cis nanogels, Cis solution of equal Pt concentration (50 μg/ml), and the corresponding mixture of HA+PB (fig. S2, B and C). While the Cis’s interaction with phosphate groups of DNA molecules was well recognized, the Pt-phos interaction within the HA nanogel conferred HA/PtP with an excellent photothermal capability.

### Structure analysis of the HA/PtP nanogels via theoretical molecular dynamics simulations

**Fig. 2.**
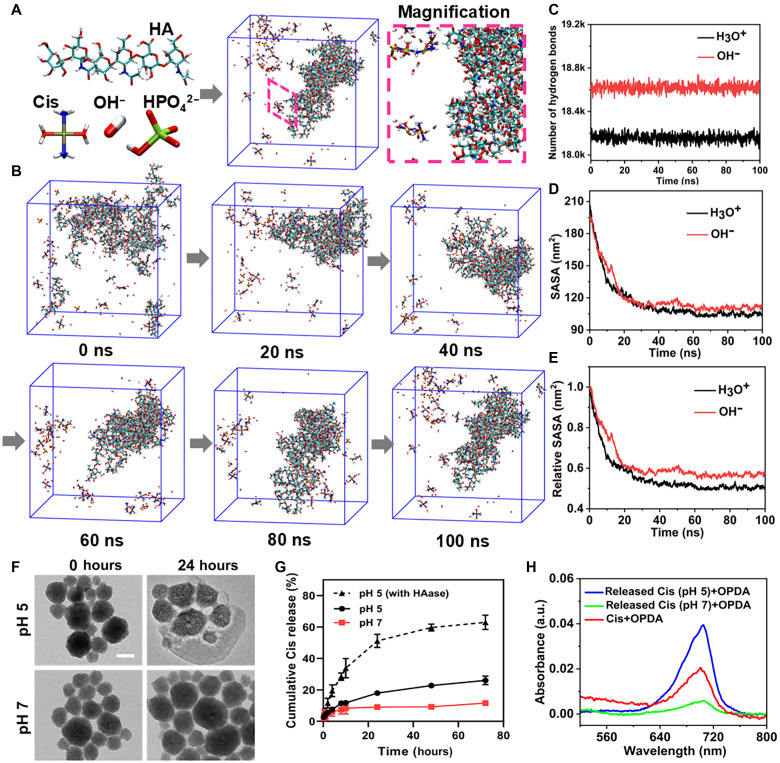
MD simulation and Cis release ability of the HA/PtP nanogels. (**A**) The snapshots of HA/PtP from MD simulation wherein the nanoparticles were zoomed up to show the structure details. (**B**) Time-dependent snapshots from MD simulation of nanogels formed by HA, Cis, and HPO4^2−^ under alkaline condition. (**C**) Total number of hydrogen bonds in an acidic/alkaline system. (**D** and **E**) The SASA and relative SASA in an acidic/alkaline system. (**F**) TEM images of HA/PtP nanogels after incubation in PBS (pH  7, 5) for 24 hours. Scale bar, 50 nm (**G**) Cumulative drug release curves of HA/PtP in PBS (pH  7, 5) and PBS (pH 5) with HAase (50 U/ml) at 37°C. (**H**) The UV-vis absorption spectra of the Cis-OPDA reaction products. After the release experiments in different pHs, the dialysates were reacted with OPDA. The peak around 705 nm is ascribed to the coordination products between Cis and OPDA.

To further probe the assembly mechanism among Cis, phosphate group, and HA propelled by intermolecular interactions, molecular dynamics (MD) simulations were performed using the GROMACS-2020.7 software package, and the molecular force field was performed using GAFF (*43*, *44*). Basically, HA to Cis to phosphate group was fed at 5:2:1, a molar ratio similar to that in HA/PtP nanogel, which was further included in a cuboid box with a length of 7 nm and subjected to 100 ns of MD simulations. Besides, pH effects were taken into consideration for the MD simulation, as the alkaline PB was noticed to promote the HA/PtP nanogels formation and enhance the absorption in the NIR region during the optimization process of nanogel preparation (figs. S13 and S14 and table S3). As shown in Fig. 2 (A and B) and movie S1, snapshots of aggregation behaviors were selected from production simulation by adding OH^−^ to simulate the environment of an alkaline solution. Under this condition, the input molecules assembled and formed the nanoparticles more rapidly in comparison with the acidic conditions that were simulated by adding the same number of H_3_O^+^ (fig. S15 and movie S2). Furthermore, the number of hydrogen bonds in HA/PtP nanogels was statistically analyzed in the MD simulation process to probe the driving force of the HA/PtP nanogels’ assembly. Because both hydrogen bond donors and acceptors exist in the structure of HA/PtP nanogels, hydrogen bond interaction might be one of the critical factors for the self-assembly of nanoparticles. As expected, the number of total hydrogen bonds between the molecular structure of HA/PtP nanogels in alkaline solution was more than that in acidic solution (Fig. 2C), as was the number of hydrogen bonds between Cis and phosphate groups (tables S4 and S5). Based on these results, the existence of the Pt-phos interaction in the structure of HA/PtP nanogels could be confirmed. Moreover, the solvent accessible surface area (SASA) of the polymers in different solutions was calculated (Fig. 2D), which was used to describe the concept of the surface area of individual atomic surfaces in a molecule that can be touched by a solvent (*45*, *46*). For comparison purposes, the SASA values were divided by their respective maximum values, resulting in the relative SASA (Fig. 2E). The calculated results indicated that the molecules under alkaline conditions could aggregate more quickly, contributing to the formation of HA/Pt nanogel with NIR absorption and excellent dispersity as observed above.

### Cis release ability of the HA/PtP nanogels

As Cis within the nanoformulations must be released to exert its cytotoxic effects for chemotherapy, the decomposition of the HA/PtP nanogel was then evaluated under an acidic pH for the following considerations. The TMEs, as well as intracellular lysosomes, are acidic and have been widely explored to mediate the pH-responsive drug release. In addition, the acidic pH has been found to hinder the assembly of HA/PtP nanogels during the preparation screening (fig. S13). The biodegradation possibility of the HA/PtP nanogels in acidic pH was directly observed by TEM images (Fig. 2F). The HA/PtP nanogels remained as homogeneous and intact spheres under neutral conditions (pH 7) after 24 hours, whereas it exhibited heterogeneous electron density under acidic conditions (pH 5), possibly due to the partial loss of Cis. As expected, the released Cis contents under acidic conditions were ~25%, much higher than under neutral conditions (Fig. 2G). This phenomenon implied the feasibility that the Pt-phos interaction weakened under acidic conditions, leading to the release of Cis. Because HA can be degraded by hyaluronidase (HAase), an enzyme that is abundant in lysosome (*47*), Cis release was also monitored in the presence of HAase at pH 5. As shown in Fig. 2G, HAase-mediated increase of Cis release was clearly found, suggesting that the HA degradation would further facilitate the Cis release from the HA/PtP. Moreover, the released Cis was still functional, since it was capable of coordinating with *O*-phenylenediamine (OPDA), as evidenced by the absorption peak around 705 nm (*8*, *48*, *49*). The peak intensity of Cis-OPDA adducts under acidic conditions is also much higher than under neutral conditions after the drug release experiment (Fig. 2H), supporting the pH-responsive release behavior of this HA/PtP nanogel. Overall, these data suggested that the Cis retained in the HA/PtP nanogels could assure the photothermal performance, and it can function as chemotherapeutics upon its release in TMEs (*29*). The local heat generation ability of HA/PtP under an 808-nm laser irradiation could accelerate the release of Cis, showing the “on-off” release properties (fig. S16). It was also considered to enhance the resultant chemotherapeutic selectivity with the light irradiation.

### Cellular uptake of the HA/PtP nanogels

**Fig. 3.**
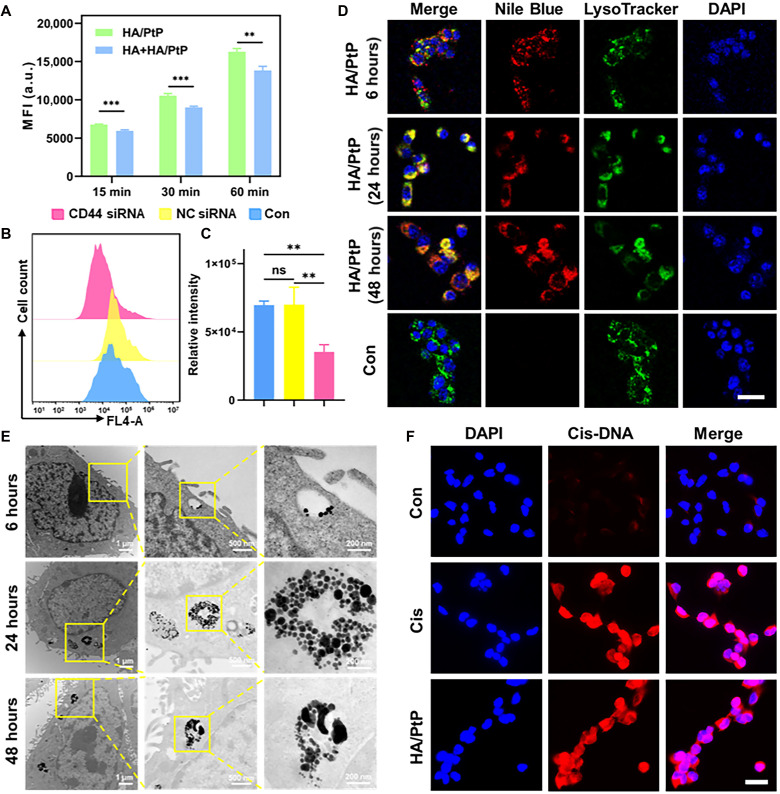
Cellular uptake of the HA/PtP nanogels. (**A**) Cellular uptake of labeled HA/PtP nanogels in 4T1 cells with or without HA competition after different incubation times (*n* = 3). (**B**) Representative FACS graphs of HA/PtP uptake in 4T1 cells after the CD44 small interfering RNA (siRNA) transfection. Con, control; NC siRNA, negative control siRNA. (**C**) Relative fluorescence intensity of cells treated by fluorescently labeled HA/PtP after the CD44 knockdown (*n* = 3). (**D**) Intracellular distribution of labeled HA/PtP nanogels in 4T1 cells after 6, 24, and 48 hours by fluorescence microscopy. Scale bar, 50 μm. (**E**) TEM images of 4T1 cells that were continuously incubated with HA/PtP nanogels for different time. (**F**) Representative images of immunofluorescence staining of Cis-DNA adducts in 4T1 cells after being incubated with HA/PtP and Cis for 12 hours. Scale bar, 25 μm. **P < 0.01; ***P < 0.001.

HA could be specifically recognized by the CD44, a surface receptor highly expressed in different cancers; thus, the CD44-targeted ability of the HA/PtP was investigated using 4T1 cells, a murine breast cancer cell line with CD44 overexpression. The HA/PtP nanogels were labeled with Nile Blue (NB) dye to allow a flow cytometry [fluorescence-activated cell sorting (FACS)] quantification. As shown in Fig. 3A, incubating the fluorescently labeled HA/PtP nanogels with extra free HA over varying time periods significantly decreased the cellular uptake. This competition assay suggested that the HA/PtP nanogels still had the active targeting ability of HA. Further knocking down the CD44 expression in the 4T1 cells was conducted (fig. S17), which clearly inhibited the cellular uptake of the fluorescently labeled HA/PtP (Fig. 3, B and C) and subsequently confirmed the CD44 targeting potential. The fluorescently labeled HA/PtP nanogels were specifically colocalized with the lysosomes after 6-, 24-, and 48-hour coincubation (Fig. 3D), as lysosomes were intracellular metabolic sites for HA after the CD44-mediated endocytosis (*50*–*52*). Because of the high electron density of Pt in HA/PtP nanogels, the subcellular localization could be determined through ultrastructural observation using cellular TEM. As shown in Fig. 3E, a large amount of HA/PtP nanogels was localized in lysosomes after incubating with 4T1 cells for 6 hours, and the amount of nanogels in lysosomes increased as the incubation time increased. After 24 hours of incubation, part of HA/PtP nanogels in lysosomes appeared in an irregular shape with lighter electron density, which is similar to that observed in acidic pH buffer (Fig. 2F), suggesting that this HA/PtP nanogel could be degraded in lysosomes. To further confirm this cellular degradability, a similar HA nanogel without a phosphate group but containing Pt nanoparticles (named as HA/Pt) was prepared by reducing the Cis in situ (fig. S18A) (*52*). Then 4T1 cells were pulsed with HA/Pt and HA/PtP nanogels for 1 hour and chased for another 24 hours (fig. S18B). As Pt nanoparticles are inert and cannot be degraded in cells, the HA/Pt could be persistently found in the lysosomes after 24 hours of incubation (fig. S18, C to E). For HA/PtP nanogel, a clearly shrunk nanogel of low electron density was found within the lysosome after 24 hours chasing, which supported the finding that the HA/PtP could decompose and release Cis with the cells. This phenomenon could be easily understood since lysosome is the degradation site of HA and its low pH would accelerate the release of Cis from HA/PtP nanogel. To confirm the release of Cis from HA/PtP nanogel within the cells, the Cis’s coordination adducts with cellular DNA were directly analyzed by immunofluorescence staining using an antibody recognizing the Cis-DNA adducts. As shown in Fig. 3F, the HA/PtP nanogels, like free Cis, could elicit a clear accumulation of Cis-DNA adducts in 4T1 cells. This evidence clearly suggested that HA/PtP nanogel could be selectively delivered to cancer cells, get degraded, and release Cis within the TMEs.

### In vitro anticancer performance of the HA/PtP nanogels

**Fig. 4.**
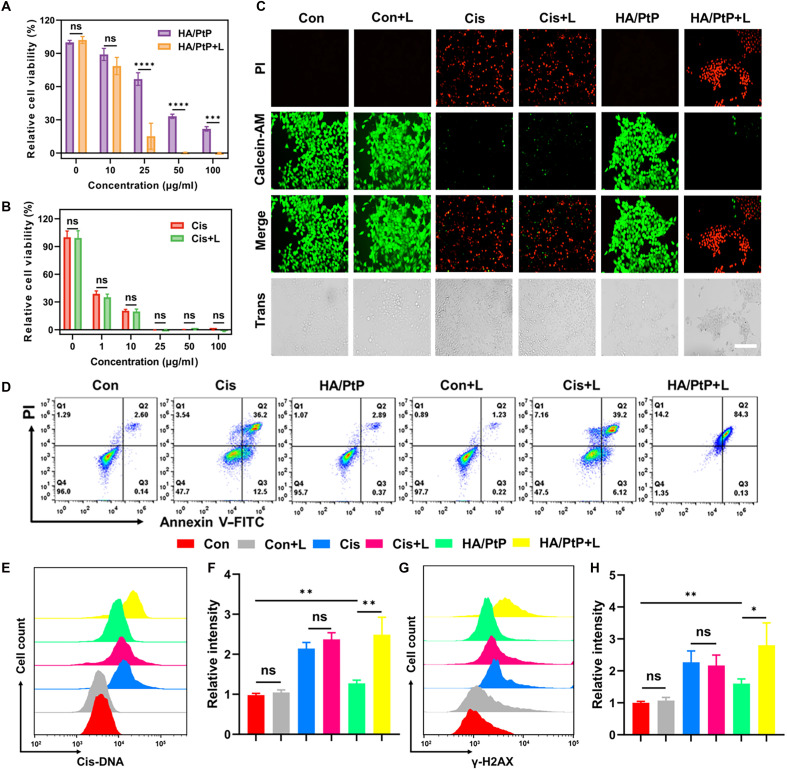
In vitro anticancer performance of the HA/PtP nanogels. (**A** and **B**) Cell viabilities of 4T1 cells incubated with HA/PtP nanogels or Cis at diverse concentration (Pt element, 0, 10, 25, 50, and 100 μg/ml) with or without an 808-nm laser irradiation (1.2 W/cm^2^, 5 min) (*n* = 3). (**C**) Fluorescence microscopy images of 4T1 cells with different treatments. The cells were stained with calcein-AM (green, live cells) and PI (red, dead cells). Scale bar, 150 μm. (**D**) Representative FACS graphs for apoptosis staining of cells after different treatments for 12 hours (*n* = 4). (**E**)The formation of Cis-DNA adducts in 4T1 cells after different treatments for 12 hours, and (**F**) corresponding relative intensity by FACS (*n* = 4). (**G**) The formation of γ-H2AX in 4T1 cells after different treatments for 12 hours, and (**H**) corresponding relative intensity by FACS. *P < 0.05; **P < 0.01; ***P < 0.001; ****P < 0.0001. ns, not significant.

The PTT/chemotherapeutic efficacy in vitro was further evaluated with or without 808-nm laser irradiation using the cell counting kit-8 (CCK-8) assay (Fig. 4, A and B). As shown in Fig. 4A, HA/PtP nanogels displayed a concentration-dependent cytotoxicity against 4T1 cells regardless of the presence or absence of laser, and they also exhibited stronger killing effects under laser irradiation. As a stark contrast, there was no obvious difference with or without laser irradiation for free Cis (Fig. 4B). The in vitro anticancer activity of the HA/PtP nanogels was also evaluated using calcein-AM/propidium iodide (PI) (live/dead) staining, which was consistent with the CCK-8 results. Upon laser irradiation, almost all 4T1 cells were killed by HA/PtP nanogels, which were competent to free Cis of the same concentration with or without laser irradiation (Fig. 4C). Furthermore, FACS with annexin V–fluorescein isothiocyanate (FITC)/PI fluorescence staining also demonstrated that the HA/PtP nanogels combined with laser irradiation exhibited the strongest apoptosis-inducing ability (Fig. 4D).

To further confirm the effects of synergistic PTT/chemotherapy in vitro, the Cis-DNA adducts in differently treated cells were quantified based on FACS. As shown in Fig. 4 (E and F), the HA/PtP nanogels could effectively induce the formation of Cis-DNA adducts by releasing free Cis. In addition, NIR irradiation promoted the formation of Cis-DNA adducts, which inferred that localized heating accelerated the release of Cis within the cell. The Cis-DNA adduct–caused lesion would induce the phosphorylation of H2AX (γ-H2AX) (*22*), and thus, γ-H2AX as a marker for Cis-related DNA damage was also evaluated. As shown in Fig. 4 (G and H), similar to free Cis treatment, the HA/PtP nanogels effectively induced H2AX phosphorylation, suggesting that they retained the chemotherapeutic function of Cis. Under NIR irradiation, the HA/PtP nanogels caused more DNA damage compared with no laser. These results collectively suggested that the synergistic PTT/chemotherapy could be acquired by simply combining the photothermal potential of HA/PtP and the chemotherapeutic activity of released Cis in vitro.

### In vivo antitumor activity of the HA/PtP nanogels

**Fig. 5.**
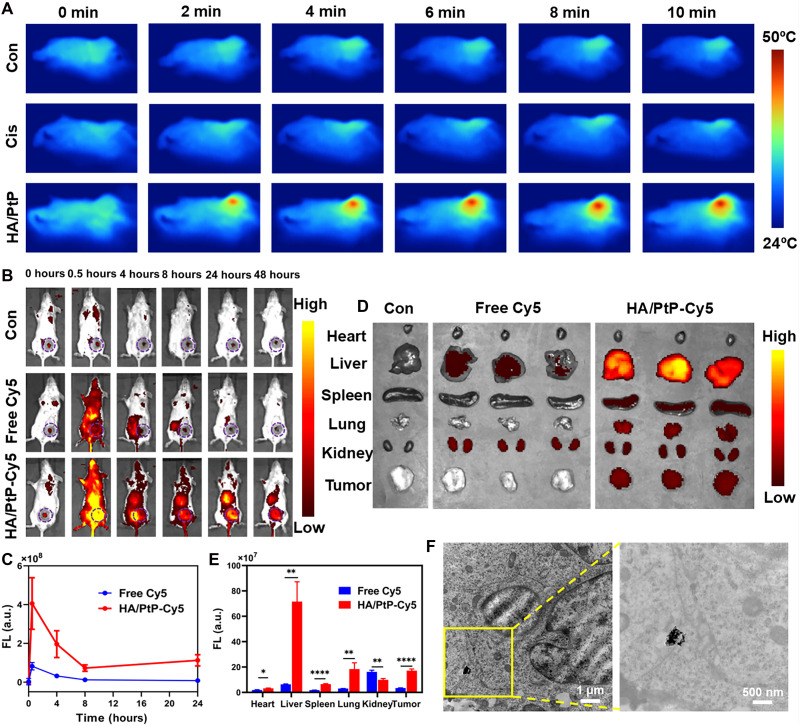
Biodistribution of HA/PtP nanogels. (**A**) Infrared thermography of 4T1 tumor-bearing mice after being injected with PBS, Cis, and HA/PtP nanogels (Pt, 5 mg/kg) under an 808-nm laser irradiation (0.8 W/cm^2^). (**B**) Representative fluorescence imaging of the 4T1 tumor-bearing mice at different time points after intravenous injection of Cy5-labeled HA/PtP nanogels (HA/PtP-Cy5) and free Cy5 (Pt, 5 mg/kg), and (**C**) corresponding quantification of the tumoral fluorescence (*n* = 3). (**D**) Ex vivo fluorescence imaging of the major organs after the systematic administration with different formulations for 48 hours, and (**E**) corresponding fluorescence intensity (*n* = 3). (**F**) TEM image of 4T1 tumor after intravenous injection of HA/PtP nanogels for 72 hours (Pt, 10 mg/kg). *P < 0.05; **P < 0.01; ***P < 0.001; ****P < 0.0001.

Encouraged by the outstanding in vitro PTT/chemotherapy anticancer performance, we further evaluated the HA/PtP nanogels antitumor effects in vivo. The distinct NIR absorbance and good photothermal stability made the HA/PtP nanogels an ideal contrast agent for thermal imaging. After systematic administration of PBS (control), Cis, and HA/PtP nanogels (5 mg/kg, Pt element) in 4T1 tumor-bearing mice for 24 hours, the temperature of the HA/PtP-treated mouse exhibited an obvious rise to 47.3°C under an 808-nm laser irradiation for 10 min (Fig. 5A and fig. S19), which would allow mild hyperthermia and minimize damage to surrounding tissues. In comparison, the temperature of PBS and Cis-treated mice only displayed a slight increase to 34.7° and 34.9°C, respectively. To validate whether the improved heat generation at the HA/PtP nanogel–treated tumor was derived from its tumor-targeted delivery, the biodistribution of the HA/PtP nanogels was performed by in vivo fluorescence imaging. The 4T1 tumor-bearing mice were imaged at different time points with PBS, free Cy5, and Cy5-labeled HA/PtP nanogels (HA/PtP-Cy5) (Fig. 5, B and C). Compared with the control and free dyes, the fluorescently labeled HA/PtP exhibited stronger intensity and longer retention of fluorescence at the tumor sites, supporting the improved in vivo delivery due to the targeting effects of HA. The tumor accumulation ability of HA/PtP was also confirmed by ex vivo imaging 48 hours postinjection. As shown in Fig. 5 (D and E), strong fluorescence signals were observed in the tumor collected from the HA/PtP-treated mice. To affirm the tumoral localization of HA/PtP nanogels in vivo, the tumor tissues of HA/PtP nanogel–treated mice were excised at 72 hours postinjection and observed by biological TEM. Compared with the control tumor (fig. S20), HA/PtP nanogels of visible electron density were clearly spotted in the tumor tissue (Fig. 5F). These data suggested that the injected HA/PtP nanogel remained in tumors after 3 days, which would allow PTT with the light irradiation.

**Fig. 6.**
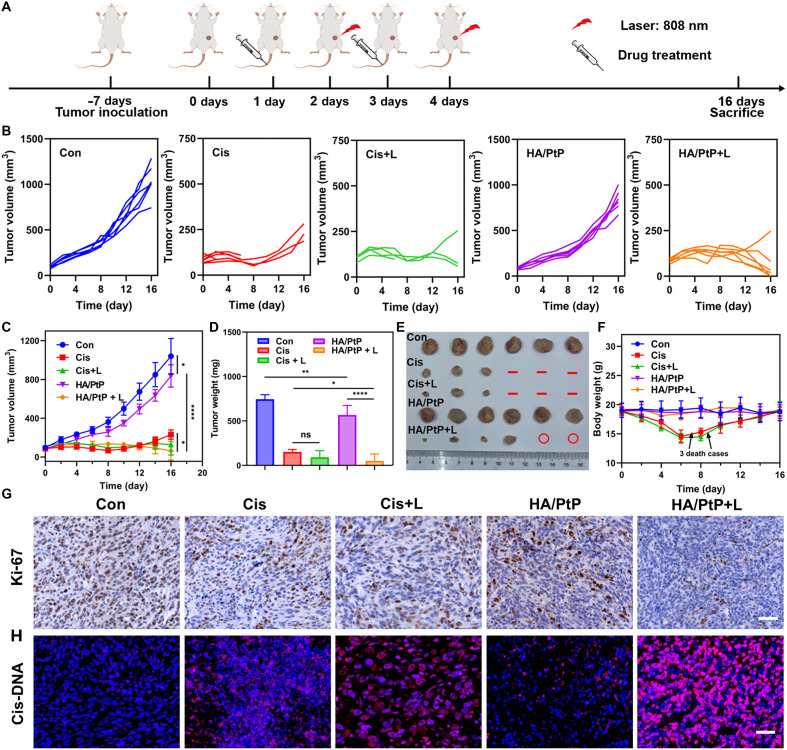
Antitumor evaluation of HA/PtP nanogels in 4T1 tumor-bearing mice model. (**A**) Experimental schedule for tumor inoculation, administration, and PTT treatments. (**B**) Tumor growth of each group. The different treatments were applied every 2 days (*n* = 6). (**C**) Tumor volume, (**D**) tumor weight, and (**E**) photograph of excised tumor. “**-**” represents mice’s death; “**○**” represents complete tumor regression (*n* = 6). (**F**) Mice body weight (*n* = 6). (**G**) Ki-67 and (**H**) Cis-DNA adducts staining of tumor tissues. Scale bars, 50 μm. *P < 0.05; **P < 0.01; ****P < 0.0001.

**Fig. 7.**
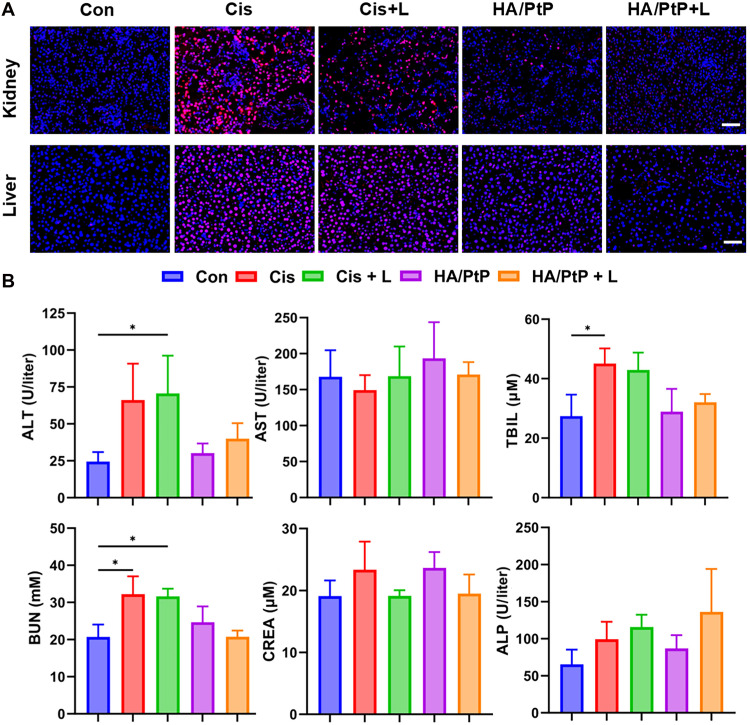
Safety evaluation of HA/PtP nanogels in vivo. (**A**) Cis-DNA adducts staining of kidney and liver tissues from 4T1 tumor-bearing mice following different treatments. Scale bars, 50 μm. (**B**) Serum biochemical analysis (*n* = 3). *P < 0.05.

Given the tumor-targeted delivery of HA/PtP nanogels, the in vivo antitumor performance was further conducted in 4T1 tumor-bearing mice following the regimen depicted in Fig. 6A. The mice were randomly divided into five groups (*n* = 6): (1) saline, (2) Cis only, (3) Cis with an 808-nm laser irradiation (Cis+L), (4) HA/PtP only, and (5) HA/PtP with an 808-nm laser irradiation (HA/PtP+L). As shown in Fig. 6 (B to F) and fig. S21, HA/PtP with NIR laser irradiation treatment outperformed the Cis treatments in reducing the tumor volume and weight, which could be due to the combined therapeutic effects of synergistic PTT/chemotherapy. Owing to the chemotherapeutic efficacy of delivered Cis, the HA/PtP only also induced a medium tumor inhibition. The corresponding staining of the tumor proliferative index marker of Ki-67 verified the significant tumor inhibition of HA/PtP with the NIR laser irradiation group (Fig. 6G). Furthermore, this tumor proliferation inhibition ability was also adequately supported by the staining of Cis-DNA adducts in the excised tumors. As shown in Fig. 6H, the strongest Cis-DNA staining signals were observed in the HA/PtP+L group than in other treated groups, which strongly suggested that the tumor-targeted delivery enabled by HA and the Cis could be released from the HA/PtP nanogel within the TMEs. Moreover, PTT would also facilitate the Cis release as more Cis-DNA adducts were observed in the case of HA/PtP+L compared with that of HA/PtP. While administration of free Cis clearly inhibited tumor growth (79.6% inhibition in tumor weight), the free Cis plus light (87.8% inhibition in tumor weight) failed to bring a significantly improved tumor inhibition. However, due to the severe side effects, three cases of mouse death were found for the free Cis treatments, which could also be reflected by the dramatically reduced mouse body weight in the cases of Cis with or without light (Fig. 6E). Then, hematoxylin and eosin (H&E) staining of the main organs including the heart, liver, spleen, lung, and kidney was also performed to confirm the toxicity profile. As shown in fig. S22, kidneys exhibited signs of renal tubular damage and vascular harm, whereas areas of inflammation were detected in both the liver and kidney when mice were treated with Cis and Cis+L. It is well documented that the kidneys and liver are the primary targets of Cis-induced toxicity (*5*). The staining of Cis-DNA adducts in kidney and liver slides also supported this phenomenon, as more Cis-DNA adducts appeared in both Cis and Cis+L groups than in other groups (Fig. 7A). Whereas for the HA/PtP groups, regardless of laser irradiation or not, less pathological variation and accumulation of Cis-DNA adducts were observed in both kidney and liver. The relieved side effects of the HA/PtP nanogel were also validated in the biochemical blood analysis. Although blood urea nitrogen (BUN) and total bilirubin (TBIL), which reflect kidney and liver dysfunction, are significantly changed in free Cis treatments (Fig. 7B), there is no significant change in the biochemical markers for HA/PtP-treated mice compared with the control mice. Hence, the above observations substantiated the finding that the HA/PtP nanogels could relieve the severe side effects that were notorious for the Cis-based chemotherapy regimen. Besides, the combination with PTT did not induce an extra toxicity profile, supporting the tolerability of synergistic PTT/chemotherapy based on the targeted delivery of HA/PtP nanogels.

### Mimicking HIPEC based on the HA/PtP nanogels

**Fig. 8.**
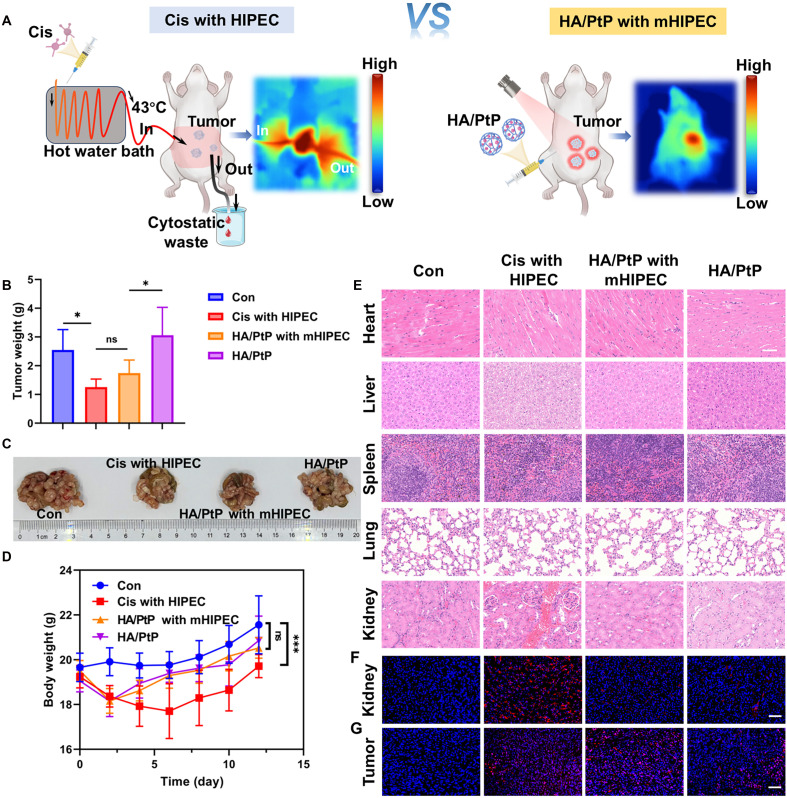
Mimicking HIPEC based on the HA/PtP nanogels. (**A**) Experimental schemes comparing the HIPEC (Cis) and mHIPEC (HA/PtP) in MC38 intraperitoneal peritoneal metastasis model and corresponding infrared thermography images. (**B**) Weight of the tumor nodules (*n* = 6). (**C**) Representative photograph of tumor burden. (**D**) Mouse body weight was recorded after different treatments (*n* = 6). (**E**) H&E staining in main organs. Scale bar, 50 μm. Cis-DNA adducts staining of (**F**) kidney and (**G**) tumor tissues. Scale bars, 50 μm. *P < 0.05; ***P < 0.001.

Cis has also been clinically applied in combination with hyperthermia for the treatment of peritoneal metastases through a procedure known as HIPEC, in addition to its conventional use in treating localized solid tumors (*32*, *33*). Following the cytoreductive surgery, a typical HIPEC protocol requires Cis to be heated to 40° to 43°C before its infusion into the peritoneal cavity (Fig. 8A). With the application of a HIPEC device to circulate the heated drug, hyperthermia would help the Cis penetration and thus effectively eliminate the metastasis. Given the role of HA/PtP as a targeted nanocarrier, PTA, and Cis formulation, the PTT based on HA/PtP nanogel was expected to mimic the HIPEC (mHIPEC) with light-dependent heat and relieved toxicity. Thus, we performed the mHIPEC using a colorectal cancer MC38 metastasis model, and the mice were methodically treated by different formulations as depicted in Fig. 8A. The mice were randomly divided into four groups (*n* = 6): (1) saline, (2) Cis with HIPEC, (3) HA/PtP with mHIPEC, and (4) HA/PtP only. Seven days post–tumor inoculation, the Cis treatment group was administered using the conventional HIPEC methods. Cis solution (0.1 mg/ml, Pt element) was heated to 43°C and infused into the abdominal cavity of mice at a flow rate of 1 ml/min for 10 min, after which the excess perfusate was drained. Throughout the procedure, the increased temperature at the abdominal cavity of mice was monitored using an NIR thermal imaging device (Fig. 8A, left). For the mHIPEC group, 2 ml of HA/PtP nanogels was administered via intraperitoneal injection (0.1 mg/ml, Pt element), and NIR laser irradiation was performed after 12 hours. During the process, the temperature of the irradiated area was controlled under 45°C by adjusting the laser power density, and monitored using an NIR thermal imaging device (Fig. 8A, right). Compared with control, both the mHIPEC with HA/PtP and HIPEC with free Cis significantly reduced the tumor nodules’ weight (Fig. 8, B and C). While tumor inhibition efficacy was comparable between these two HIPEC groups, HA/PtP with mHIPEC demonstrated an NIR laser–dependent tumoral inhibition compared with the HA/PtP-only group, along with reduced body weight loss in reference to Cis with HIPEC (Fig. 8D). These data suggested both therapeutic safety and spatiotemporal controllability of the mHIPEC based on HA/PtP. The similar antitumor effects of mHIPEC and HIPEC in the metastasis model could be attributed to two factors: (i) the substantially lower Cis dosage involved in HA/PtP with mHIPEC (0.2 mg Pt per mouse) versus Cis with HIPEC (1 mg Pt perfusion per mouse) and (ii) incomplete photothermal ablation of deeply embedded peritoneal nodules (e.g., those beneath intestinal structures) due to inherent light penetration limitations in phototherapy. To further compare the biosafety of this mHIPEC strategy, the H&E staining of main organs was conducted. As expected, Cis with HIPEC treatment exhibited clear damage in both liver and kidney (*53*), while no significant abnormalities were observed in the groups treated with HA/PtP (Fig. 8E). The staining of Cis-DNA adducts in kidney slides also showed more Cis-DNA adducts in the Cis with HIPEC group (Fig. 8F). Simultaneously, clear accumulation of Cis-DNA adducts was observed at the tumor site of the HA/PtP with mHIPEC treatment (Fig. 8G). The widely used HIPEC strategy relies on a specialized hyperthermic perfusion device to circulate heated Cis within the abdominal cavity, facing critical limitations including imprecise temperature control and dose-dependent nephrotoxicity. In contrast, the HA/PtP with mHIPEC method mediates spatiotemporally controlled photothermal activation through NIR irradiation, and TME-responsive Cis release that synergizes with PTT to minimize systemic toxicity, particularly the kidney damage that is commonly associated with free Cis (*53*).

**Fig. 9.**
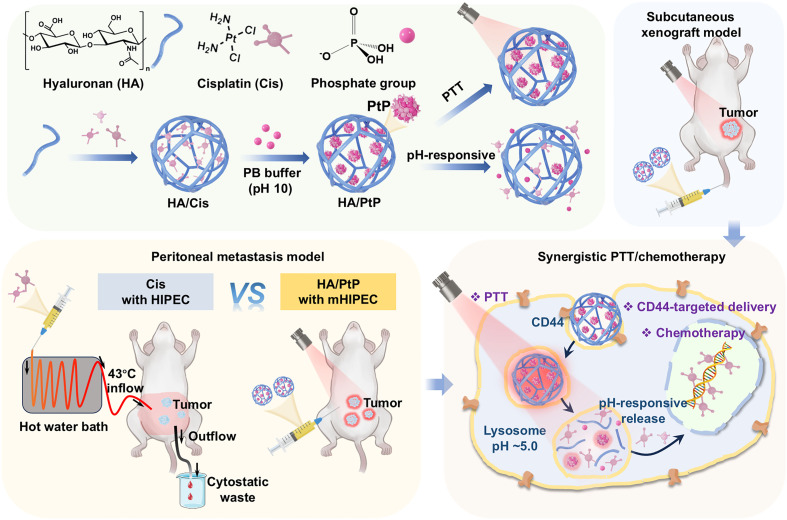
Schematic illustration of the construction of HA/PtP nanogels and the proposed mechanism for their anticancer applications. HA/PtP features self-targeting that is inherent to HA, PTT effects enabled by PtP, and a pH-responsive release of Cis within the TMEs, allowing a synergistic PTT/chemotherapy for both subcutaneous xenograft and peritoneal metastasis models.

To summarize, HA/PtP nanogel with advanced photothermal performance and tumor-targeting ability was successfully fabricated to enable synergistic PTT/chemotherapy. The HA/PtP nanogels were synthesized through a simple self-assembly method inspired by a Pt-phos interaction, which contributes to the stabilization of Cis-DNA adducts. While the Pt-phos form in HA/Pt nanogel allowed PTT, it can be reversed and release Cis within the TMEs and thus enable the targeted chemotherapy. To the best of our knowledge, no prior studies have reported the dual-functional transformation of Cis into a PTA while maintaining its inherent chemotherapeutic activity within the TMEs. The HA could specifically recognize CD44, which is highly expressed on the surface of various cancer cells, thereby endowing HA/PtP nanogels with good cancer cell–targeting capability. Under NIR laser irradiation, the HA/PtP treatment efficiently inhibited the tumor of 4T1 breast cancer. The application of this HA/PtP could also be extended to the mHIPEC for tumor metastasis treatment (Fig. 9). The application of HA/PtP significantly reduced the toxic side effects of Cis chemotherapy. While the HA/PtP provided a promising PTT/chemotherapeutic agent, this study has several limitations that should be considered. In our case, the chemotherapy is dependent on the release of Cis, which inevitably compromise the light absorption and, subsequently, the photothermal conversion capability of HA/PtP. Thus, the timing of laser irradiation to initiate the PTT as well as achieve an optimal chemotherapeutic effect should be carefully considered. Under the current conditions, the tumor inhibition efficiency of HA/PtP-based mHIPEC for the colorectal cancer metastasis model was comparable to that of Cis HIPEC. As a light therapy, the penetration depth of PTT remains limited, particularly in human tissues, which is another important consideration for potential clinical translation. In the future, optimization of mHIPEC technology such as fine-tuning the timing and dosage of HA/PtP treatment and using light fiber or NIR-II light sources would further improve the PTT/chemotherapy efficacy for peritoneal metastases management. Overall, these HA/PtP nanogels might offer great promise for synergistic PTT/chemotherapy.

## MATERIALS AND METHODS

### Materials

Hyaluronate (HA; molecular weight = 90 to 100 kDa) was purchased from Yuanye Bio-Technology Co., Ltd. (Shanghai, China). Cis was obtained from Macklin Biochemical Technology Co., Ltd. (Shanghai, China). Disodium hydrogen phosphate and sodium phosphate monobasic dihydrate were bought from Aladdin (Shanghai, China). *N*-hydroxysuccinimide (NHS), *N*-(3-dimethylaminopropyl)-*N*′-ethylcarbodiimide hydrochloride crystalline (EDC), HAase (no. H3506), OPDA, and dimethyl sulfoxide were purchased from Sigma-Aldrich (St. Louis, USA). CCK-8 and annexin V–FITC apoptosis detection kit were purchased from Dojindo Molecular Technologies (Kumamoto, Japan). Hoechst 33342 (no. 15547) was bought from Cayman Chemical Company (Ann Arbor, MI, USA). LysoTracker Deep Red (L12492) was bought from Life Technologies (New York, NY, USA). RPMI 1640 cell culture medium and fetal bovine serum (FBS) were purchased from Thermo Fisher Scientific (Waltham, MA, USA). All other chemical agents were analytical grade and obtained from various commercial sources.

### Synthesis of HA/PtP and HA/Cis nanogels

Cis (15 mg/ml) was immersed in deionized water and heated to 90°C until it was fully dissolved. HA (5 mg/ml) was dissolved in deionized water. Then 200 μl of Cis was mixed with 400 μl of HA and heated at 90°C. After 5.5 hours heating at 90°C, 400 μl of PB in different pH was added into the above mixture and blended completely. The obtained mixture was heated at 90°C until the color of the solution no longer changed. Last, the HA/PtP nanogels were collected by centrifugation and subjected to complete dialysis in 1 liter of deionized water for 24 hours. Meanwhile, the HA/Cis nanogels were prepared in the same process without adding PB. To label the HA/PtP nanogels, 25 μl of NB in *N*,*N*′-dimethylformamide (DMF; 2 mg/ml) was introduced into the above mixture before the addition of HA. The quantification of Cis in nanogels is characterized by inductively coupled plasma mass spectrometry (ICP-MS; Agilent 5110, Agilent Technologies, USA) in the determination of Pt element’s content. To label HA/PtP with Cy5, 40 mg EDC and 8.5 mg NHS were added to 1 ml of HA/PtP (1 mg/ml) and stirred for 30 min to activate the carboxylic groups in dark at room temperature. Then, 1 ml of Cy5-NH_2_ (Lumiprobe GmbH, Germany) in DMF (1 mg/ml) was introduced into the above mixture and stirred for 24 hours in dark at room temperature. Last, the Cy5-labeled HA/PtP nanogels were collected by dialysis in 1 liter of deionized water for 24 hours. The quantification of Cy5 in nanogels is characterized based on the fluorescence using a microplate reader (Thermo Fisher Scientific, Waltham, MA, USA).

### Characterization of HA/PtP nanogels

TEM and STEM images of HA/PtP nanogels were observed on a TEM microscope (JEM-F200, JEOL, Japan). Five microliters of diluted HA/PtP was added onto a copper grid and stained by uranyl acetate (1.5% in ddH_2_O) for the TEM sample preparation. The 4T1 tumor and cell samples with different treatments were fixed with 2.5% glutaraldehyde, subjected to a standard protocol for the preparation of ultrathin sections and then observed by a TEM microscope (JEM-1400Plus, JEOL, Japan). SEM images and SEM-EDS spectra were obtained on a SEM microscope (JSM7610F, Hitachi, Japan). The hydrodynamic diameter in PBS or deionized water and zeta potential of HA/Pt nanogels were determined using the Malvern Zetasizer Nano ZS90 machine (Malvern Instruments, Malvern, UK). All measurements were conducted at room temperature. The concentrations of Cis, HA/Cis, HA/Pt, and HA/PtP in this study were standardized based on Pt content as quantified by ICP-MS (7800MS, Agilent, USA). The absorption spectra of different nanogel aqueous solutions were recorded on a UV-vis spectrophotometer (UH5300, Hitachi, Japan). FTIR spectroscopy analysis was conducted on an FTIR spectrometer (Nicolet iS20, Thermo Fisher Scientific, USA) in the range of 4000 to 400 cm^−1^. XPS spectra of HA/PtP nanogels, Cis, and PB (pH 10) were conducted using the Thermo Fisher Scientific K-Alpha XPS instrument (Thermo Fisher Scientific, USA). For XRD analysis, lyophilized HA/PtP nanogel was subjected on a Rigaku SmartLab SE diffractometer (Rigaku, Japan). The TG analysis with a heating rate of 15°C/min from room temperature to 1000°C in a N_2_ atmosphere was demonstrated using Rigaku TG/DTA 8122 (Rigaku, Japan).

### Photothermal performance of HA/PtP nanogels

One milliliter of HA/PtP nanogel aqueous solutions with different concentrations of Pt element (0, 5, 10, 25, and 50 μg/ml) was added in a quartz cuvette and irradiated with an 808-nm laser at a power of 0.8 W/cm^2^ for 10 min. The temperature was recorded by an OMEGA four-channel data logger thermometer (RDXL4SD, Omega, Taiwan) at different time durations.

### DFT calculations

The DFT calculations for HA/PtP, Cis, and phosphate salt were performed with the assistance from Scientific Compass (www.shiyanjia.com). All quantum chemical computations were conducted using Gaussian 16 A.03 software. Geometry optimizations and vibrational frequency analyses were performed with the ma-def2-SVP basis set. Subsequently, single-point energy evaluations were carried out at the ma-def2-TZVPP level. Throughout this work, the PBE0 functional combined with Grimme’s DFT-D3(BJ) empirical dispersion correction was used. Wave function analyses were processed using the Multiwfn code.

### In vitro drug release of HA/PtP nanogels

The release of Cis from HA/PtP nanogel was measured by engaging a dialysis bag (molecular weight cutoff of 3.5 kDa; Thermo Fisher Scientific, Waltham, MA, USA). One milliliter of HA/PtP aqueous solution was separately sealed in a dialysis bag and immersed in 100 ml of PBS at pH 7.4 and 5 and then conducted in an oscillator at 100 rpm and 37°C. One milliliter of PBS was collected periodically, and an equal volume of fresh PBS was replenished. To evaluate the HAase-responsive release of HA/PtP, the nanogel was dispersed in PBS (pH 5) containing HAase (50 U/ml). The release content of Cis was then characterized by ICP-MS. The cumulative release ratio of Cis was calculated as follows

$$\text{Cumulative}\;\text{Cis}\;\text{release ratio}\;\left(\%\right)=\frac{1\times \sum_{i=1}^{n-1}\mathrm{Ci}+60\times \mathrm{Cn}}{\;\text{Cis}\;\text{content in nanogels}\;}\times 100\%$$ (1)

### MD simulation

The formation of HA/PtP in solution was studied using MD simulations. The composition of each simulation system is listed in table S6 (NaCl used to neutralize the net charge of the system is not shown). Simulations were performed using the GROMACS-2020.7 software package, and the molecular force field was performed using GAFF. The results were visualized using the visualization software VMD.

The nonbonding interactions contain van der Waals and electrostatic interactions, both of which have a cutoff radius set to $r_{c}=1.2$ nm. The interaction between particles is expressed using the LJ potential

$$V_{\mathrm{LJ}}=\left\{\begin{array}{c} 4\varepsilon \left[{\left(\frac{\sigma }{r}\right)}^{12}-{\left(\frac{\sigma }{r}\right)}^{6}\right]\\ 0,\;r>r_{c} \end{array}\right.,r<r_{c}$$ (2)

The electrostatic interaction between particles is expressed as the Coulomb potential

$$V_{\text{Coulomb}}=\left\{\begin{array}{c} \frac{1}{4{\pi \varepsilon }_{0}}\frac{q_{i}q_{j}}{\varepsilon_{r}r},r<r_{c}\\ 0,r\geq r_{c} \end{array}\right.$$ (3)

First, energy minimization was performed using the steepest descent method with energies and forces of 0.01 kJ/mol and 1000 kJ/mol/nm. Next, MD was run for 125,000 steps of 1 fs, which is a constant volume process using a Berendsen thermostat. MD integrates Newton’s equations using the velocity-Verlet algorithm. Then MD is run for 250,000 steps of 2 fs, using Berendsen for both the thermostat and pressure coupling. Last, MD is run for 5,000,000 steps of 2 fs, using Nose-Hoover and Parrinello-Rahman for the thermostat and pressure coupling, respectively.

### Cell culture

Murine breast carcinoma cell 4T1 and murine colon cancer cells MC38 were provided by the Cell Resource Center of Chinese Academy of Medical Sciences and Peking Union Medical College (Beijing, China). The cells were cultured in the incubator (HERA CELL 150, Thermo Fisher Scientific, USA) at 37°C with a humidified atmosphere containing 5% CO_2_. The medium contained streptomycin (100 U/ml), penicillin (100 U/ml; PS, Gibco BRL, Grand Island, NY, USA), and 10% FBS (Gibco BRL, Grand Island, NY, USA) in RPMI 1640 (Hyclone, Logan, UT, USA).

### Cellular uptake of the nanogel

The 4T1 cells were seeded in 24-well plates at 2 × 10^4^ cells per well and incubated overnight. The medium was replaced by NB-labeled HA/PtP nanogels and incubated for 6, 24, and 48 hours. Then, LysoTracker Deep Red and Hoechst 33342 were applied to stain the lysosomes and nuclei, respectively. The cells were observed with a fluorescence microscope (EVOS, Life Technologies, New York, NY, USA) through Cy5, green fluorescent protein, and 4′,6-diamidino-2-phenylindole (DAPI) channels. To identify the CD44 targeting ability of HA/PtP nanogels, cellular uptake of NB-labeled HA/PtP nanogels was assessed in the presence of additional free HA (1 mg/ml) for 2 hours. The 4T1 cells were then incubated with NB-labeled HA/PtP nanogels for 15, 30, and 60 min. The cellular fluorescence intensity was measured by FACS (BD Accuri C6, USA) in the FL4 channel.

### CD44 knocking down analysis

Small interfering RNA (siRNA) sequences targeting mouse CD44 (5′-CCAGCAAGUCUCAGGAAAUTT-3′) and a negative control (5′-UUCUCCGAACGUGUCACGUTT-3′) were synthesized by GenePharma (Shanghai, China). The transfection procedure was performed as follows: First, 4T1 cells were seeded into 12-well plates (1 × 10^5^ cells per well) and allowed to adhere overnight. Subsequently, transfection was carried out using Lipofectamine 3000 reagent (Thermo Fisher Scientific, L3000001) with 60 pmol of siRNA per well. Following 12 hours of transfection, the medium was changed to fresh medium for continued culture. The knockdown efficiency of CD44 in 4T1 cells was verified via FACS using a CD44-APC (allophycocyanin) antibody (BioLegend, catalog no. 103012). Last, these cells were treated with NB-labeled HA/PtP nanogels for 30 min prior to FACS (BD Accuri C6, USA) in the FL4 channel.

### Cytotoxicity measurement

CCK-8 assay was introduced to study the cell cytotoxicity of HA/PtP and Cis. The 4T1 cells were first seeded in 96-well plates at a density of 2 × 10^4^ cells per well and incubated overnight. Then, the cells were treated with HA/PtP and Cis at different concentrations of Pt element (0, 10, 25, 50, and 100 μg/ml) and cultured for 12 hours. After incubation, the culture medium was replaced with fresh culture medium. Then, the cells were irradiated for 5 min per well with an 808-nm laser (1.2 W/cm^2^) and incubated overnight. After that, cells were rinsed once with PBS. Then, 10 μl of CCK-8 (in 110 μl of RPMI) was added to each well, and the cells were further treated for 2 hours. Last, the absorbance at 450 nm of cells in each well was detected by a microplate reader (Thermo Fisher Scientific, Waltham, MA, USA) to determine the cell viability.

### Cell apoptosis assay

The 4T1 cells were first seeded in 24-well plates at a density of 5 × 10^5^ cells per well and incubated overnight for cell adhesion. Then, the cells were treated with medium only, Cis (1 μg/ml, Pt element), and HA/PtP nanogels (25 μg/ml, Pt element) for 12 hours. After incubation, the culture medium was replaced with fresh culture medium. Then, the cells were irradiated for 5 min per well with an 808-nm laser (1.2 W/cm^2^) and incubated overnight. For live/death evaluation of cells, calcein-AM and PI were added to the medium and incubated for 10 min to stain the cells. Then, the fluorescence images of the treated cells were acquired under a fluorescence microscope (EVOS, Life Technologies, New York, NY, USA). To quantitatively determine cell apoptosis and death, FACS analyses of 4T1 cells with same treatments were further investigated. The apoptosis detection kit was then used to treat the cells after laser irradiation. Subsequently, apoptosis was analyzed by FACS (BD Accuri C6, USA).

### FACS analysis of Cis-DNA and γ-H2AX

The 4T1 cells were first seeded in 24-well plates at a density of 1.2 × 10^5^ cells per well and incubated overnight for cell adhesion. Then, the cells were treated with medium only, Cis (1 μg/ml), and HA/PtP nanogels (25 μg/ml) for 12 hours. After incubation, the culture medium was replaced with fresh culture medium. Then, the cells were irradiated for 5 min per well with an 808-nm laser (1.2 W/cm^2^) and incubated overnight. Afterward, the cells were collected and treated with True-Nuclear Transcription Factor Buffer Set (no. 424401, BioLegend, USA). Primary antibodies, Cis-DNA antibody (no. ab103261, Abcam, UK), and phospho-histone H2AX antibodies (no. 9718S, Cell Signaling Technology, USA) were applied at room temperature for 1 hour. Then, the cells were washed with Perm buffer and incubated with fluorescently labeled secondary antibodies on ice for 30 min, followed by Perm buffer and PBS wash once, respectively. Last, the cells were resuspended in PBS and analyzed by FACS (BD Accuri C6, USA).

### Immunofluorescence assay

The 4T1 cells were first seeded on glass coverslips in 24-well plates at a density of 5 × 10^4^ cells per well and incubated overnight for cell adhesion. The cells were treated with medium only, Cis (1 μg/ml), and HA/PtP nanogels (50 μg/ml) for 24 hours. The content of the Pt element was 1 and 50 μg/ml, respectively. After incubation, the culture medium was sucked out, and cells were rinsed twice with PBS. Then, cells were fixed with 4% paraformaldehyde in PBS for 15 min at room temperature, rinsed with PBS three times, permeabilized with 0.5% Triton X-100 for 20 min at room temperature, and blocked with 5% bovine serum albumin in PBS for 30 min to hinder nonspecific binding of antibodies. Primary antibodies and Cis-DNA antibody (no. ab103261, Abcam, UK) incubation was conserved at 4°C overnight. On the next day, cells were washed three times with phosphate-buffered saline with Tween 20 (PBST) and incubated with APC-labeled secondary antibodies at room temperature for 2 hours and then washed with PBST three times. Slides were prepared using a mounting medium containing DAPI (no. s2110, Solarbio, China). The cells were then observed with the fluorescence microscope (DMi8, Leica, Germany).

### Animal studies

Female Balb/c and C57BL/6J mice of 6 weeks old were purchased from SPF Biotechnology Co., Ltd. (Beijing, China). Mice were housed under specific pathogen-free conditions. All animal experiments were performed on the basis of the National Research Council’s Guide for the Care and Use of Laboratory Animals, and the protocol was approved by the Institutional Animal Care and Use Committee of Chinese Academy of Medical Sciences and Peking Union Medical College (ACUC-XMSB-2025-019).

### In vivo fluorescence imaging

The 4T1 murine breast cancer model was established in 6-week-old female Balb/c mice by subcutaneous inoculation of 1 × 10^5^ cells into the right mammary fat pad. Tumor-bearing mice were randomized for treatment when tumors reached ~200 mm^3^ in volume. PBS, free Cy5, and Cy5-labeled HA/PtP nanogels (HA/PtP-Cy5) were administered through tail vein injection. At 48 hours postinjection, in vivo biodistribution was assessed using the IVIS Spectrum imaging system (PerkinElmer, USA). Following imaging, mice were euthanized for ex vivo fluorescence analysis of tumors and major organs (heart, liver, spleen, lungs, and kidneys).

### In vivo antitumor effects

Female Balb/c mice (6 weeks old) were introduced to established 4T1 tumor model. PBS (100 μl) containing 1 × 10^5^ 4T1 cells were subcutaneously injected into the mouse milk fat pad. When the tumor sizes increased to 50 to 80 mm^3^ (*V* = width^2×^length/2), the mice were discretionarily divided into five therapy groups (*n* = 6) with different treatments, i.e., (1) saline, (2) Cis only, (3) Cis with an 808-nm laser irradiation (0.8 W/cm^2^) for 10 min, (4) HA/PtP only, and (5) HA/PtP with an 808-nm laser irradiation (0.8 W/cm^2^) for 10 min. The involved Pt element content was 10 mg/kg. The detailed treatment options are as follows: On day 1 and day 3, the drugs were injected through the caudal vein according to the aforementioned group; on day 2 and day 4, the tumors in group 3 and group 5 were treated an 808-nm laser irradiation (0.8 W/cm^2^) for 10 min. The temperature of the tumor site was recorded by an infrared thermometer (TiS60+, Fluke, USA). After treatments, the tumor volume and the body weight of mice were measured every 2 days. Three mice were found dead on day 8 in group 2 and group 3. On day 16, mice were euthanized, and their organs and tumor tissues were collected. Furthermore, the tumor tissues and primary organs were sliced and stained for H&E, Ki-67 (GB111499, Servicebio, China), and Cis-DNA (no. ab103261, Abcam, UK) immunofluorescence analysis. Moreover, the serum samples were collected for serum biochemical analysis to analyze the levels of alkaline phosphatase (ALP), alanine aminotransferase (ALT), aspartate aminotransferase (AST), TBIL, creatinine (CREA), and BUN.

### Mimicking HIPEC

MC38 tumor model was established with female C57BL/6J mice (6 weeks old). PBS (100 μl) containing 1 × 10^6^ MC38 cells were injected into the mouse abdominal cavity. After 1 week, the mice were randomly divided into four therapy groups (*n* = 6) with different treatments, i.e., (1) saline, (2) Cis with standard HIPEC, (3) HA/PtP with mHIPEC, and (4) HA/PtP only. The detailed treatments were as follows: On day 8, the mice in group 2 were anesthetized, and two 22-gauge catheters were placed through the lateral flanks of the abdomen. The inflow catheter was inserted perpendicularly into the left hypochondrium via a small incision as illustrated in Fig. 8A, while the outflow line was positioned on the opposite side to enable continuous perfusion. The inflow catheter was immersed in a hot water bath to allow the Cis to heat to ~43°C, and the heated Cis solution (0.1 mg/ml, Pt element) was administered intraperitoneally at a flow rate of 1 ml/min for 10 min. The outflow perfusate containing unabsorbed Cis was collected as cytostatic waste through the outflow catheter. Last, the wounds were sutured closed after the catheter removal. For group 3, HA/PtP nanogels dispersed in saline were administered by a single intraperitoneal injection at day 7 (0.2 mg Pt per mouse). After 12 hours of HA/PtP administration, the abdominal skin of the mice was incised to expose the abdominal wall, and the peritoneal cavity area was irradiated by an 808-nm laser for 10 min (0.8 W/cm^2^). The skin incision was subsequently closed with sutures. To ensure experimental parallelism, mice in group 4 were treated by the same procedure in group 3 but without laser irradiation. For all the treatments, the temperature of the mouse abdomen area was monitored by an infrared thermometer (TiS60+, Fluke, USA). On day 21, mice were euthanized, and their organs and tumor tissues were collected. Furthermore, the tumor tissues and main organs were sliced and stained for H&E and Cis-DNA (no. ab103261, Abcam, UK) immunofluorescence analysis.

### Statistical analysis

All data were expressed as means ± standard deviation (SD). One-way analysis of variance (ANOVA) or Student’s *t* test was used for the results. The differences were considered statistically significant when *P < 0.05, **P < 0.01, ***P < 0.001, and ****P < 0.0001.
