# Supplementary material for: Transforming cisplatin into targeted photothermal chemotherapeutics through the platinum-phosphate coordination within a hyaluronan nanogel
Source: Sci Adv. 2026 Mar 18;12(12):eadz7615. doi: 10.1126/sciadv.adz7615 (PMC12998518; doi:10.1126/sciadv.adz7615)
Supplement: Supplementary file 1 — Figs. S1 to S22 Tables S1 to S6 Legends for movies S1 and S2 [file sciadv.adz7615_sm.pdf]

Science Advances
AAAS

Supplementary Materials for

Transforming cisplatin into targeted photothermal chemotherapeutics through the platinum-phosphate coordination within a hyaluronan nanogel

Yiyi Zhang *et al.*

Corresponding author: Weiqi Zhang, zwq@ibms.pumc.edu.cn

Sci. Adv. **12**, eadz7615 (2026)
DOI: 10.1126/sciadv.adz7615

The PDF file includes:

Figs. S1 to S22
Tables S1 to S6
Legends for movies S1 and S2

Other Supplementary Material for this manuscript includes the following:

Movies S1 and S2

Supplementary

Text Figures

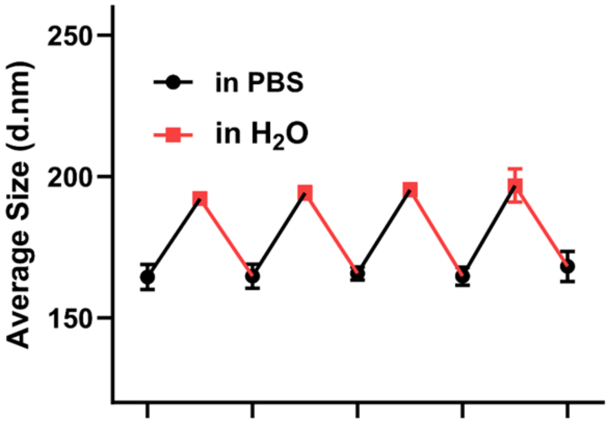

Fig.S1. Hydrodynamic size change of HA/PtP. Solution of HA/PtP nanogel was sealed in dialysis tube (MWCO=3.5 kDa) and incubated alternately in PBS (pH=7) and deionized water.

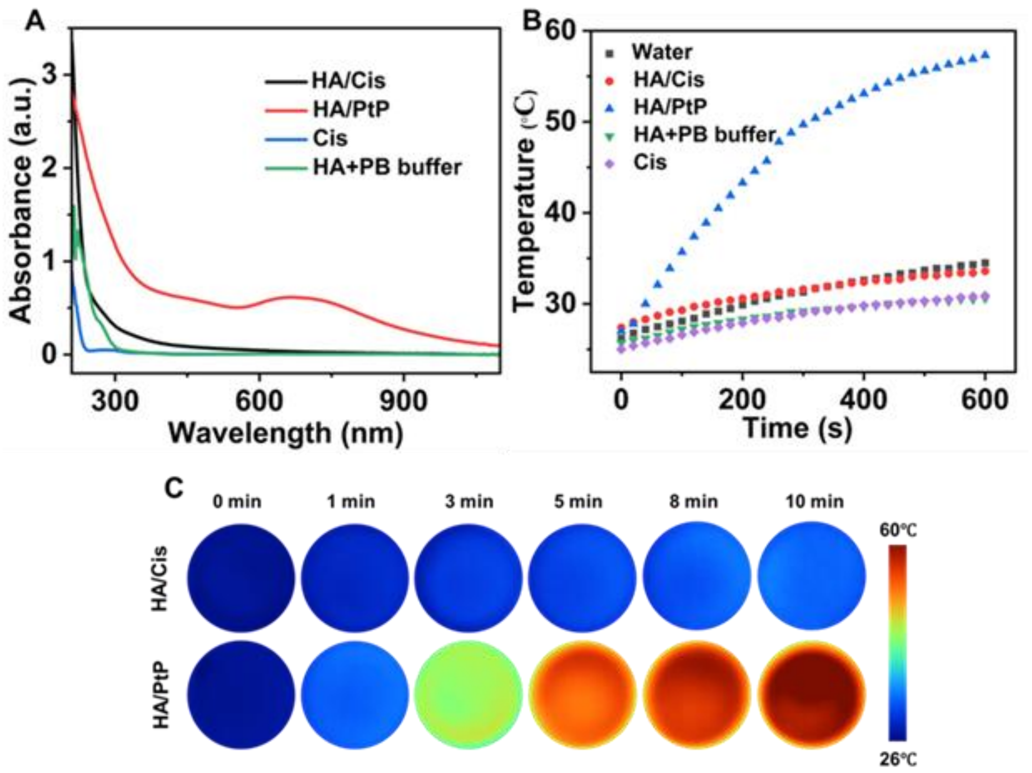

Fig. S2. UV-vis absorption and photothermal performance of different components. (**A**) UV-vis absorption spectra and (**B**) photothermal heating curves of HA/Cis, HA/PtP, Cis and HA+PB buffer aqueous solutions (Pt element, 50 μg/mL) under an 808 nm laser irradiation (0.8 W/cm^2). (**C**) Infrared thermography images of HA/Cis and HA/PtP solutions (Pt element, 50 μg/mL) under an 808 nm laser irradiation (0.8 W/cm^2).

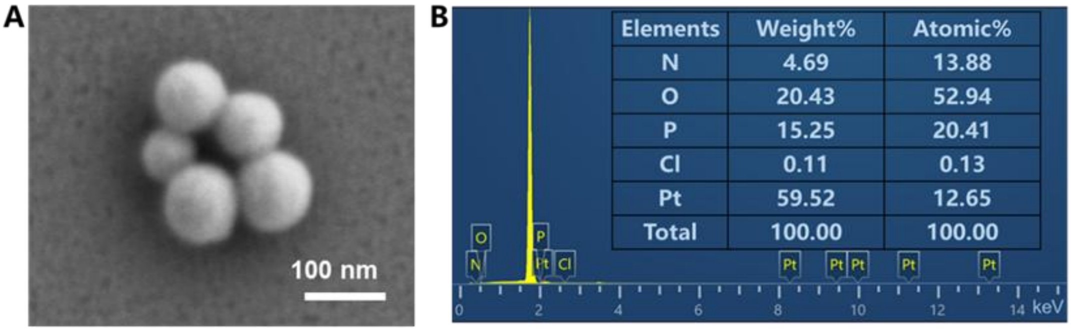

Fig. S3. SEM imaging and elemental composition of HA/PtP nanogels. (**A**) SEM images of HA/PtP nanogels. (**B**) SEM-EDS spectra and the corresponding element compositions of HA/PtP nanogels.

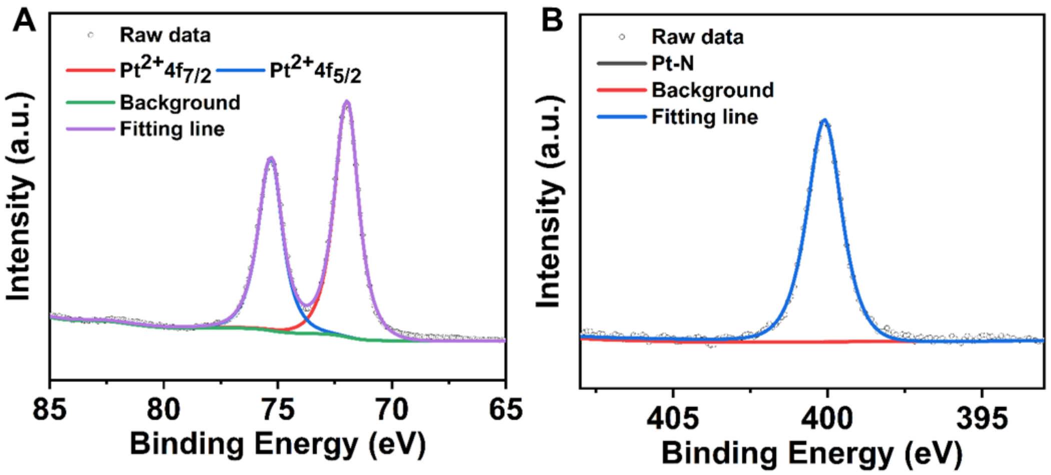

Fig. S4. High-resolution XPS analysis of Cis. High-resolution (**A**) Pt 4f, (**B**) N 1s XPS spectra of Cis.

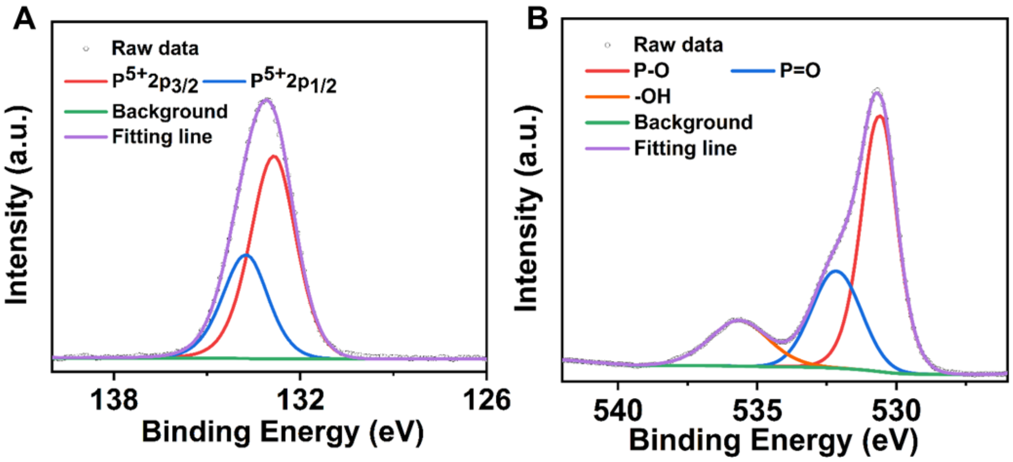

Fig. S5. High-resolution XPS analysis of PB buffer. High-resolution (**A**) P 2p, (**B**) O 1s XPS spectra of PB buffer (pH=10).

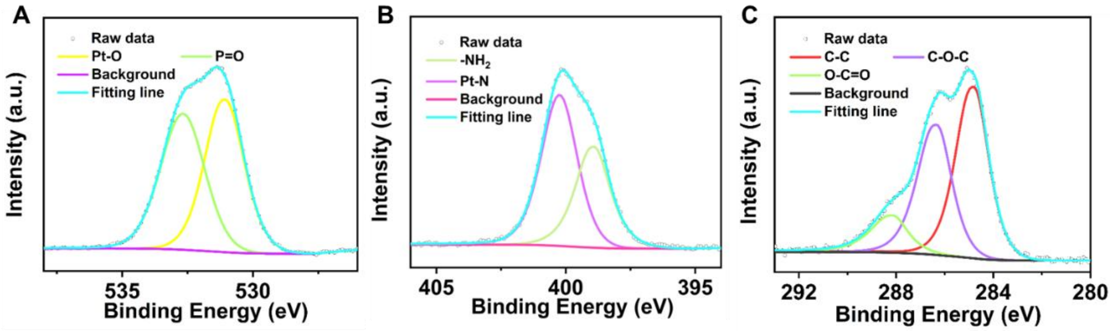

Fig. S6. High-resolution XPS analysis of HA/PtP nanogels. High-resolution (**A**) C 1s, (**B**) N 1s and (**C**) O 1s XPS spectra of HA/PtP nanogels.

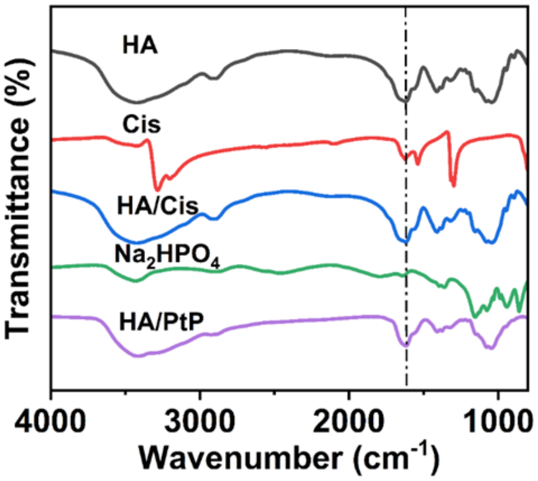

Fig. S7. FTIR spectra of HA, Cis, HA/Cis, Na_2HPO_4 and HA/PtP.

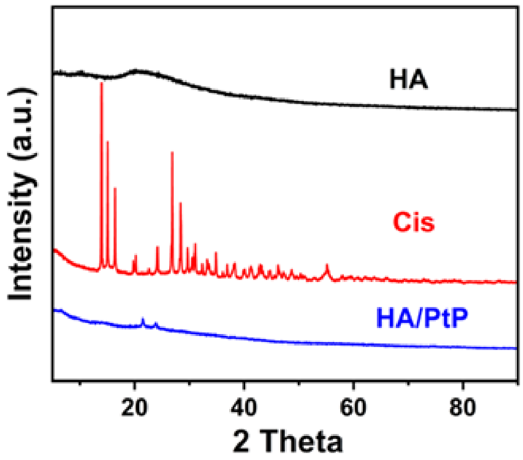

Fig. S8. XRD spectra of HA, Cis, and HA/PtP.

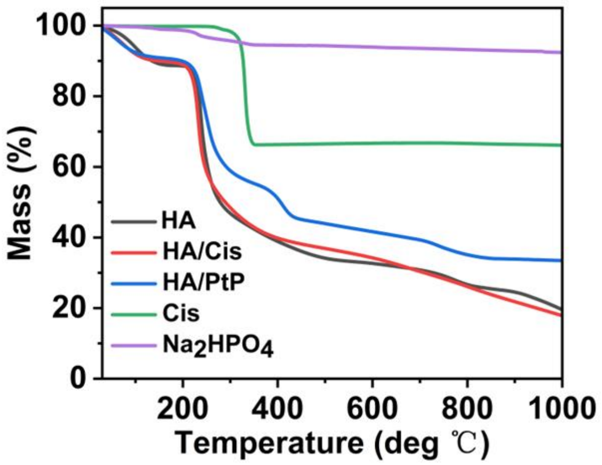

Fig. S9. TGA curves of HA, Cis, HA/Cis, Na_2HPO_4 and HA/PtP.

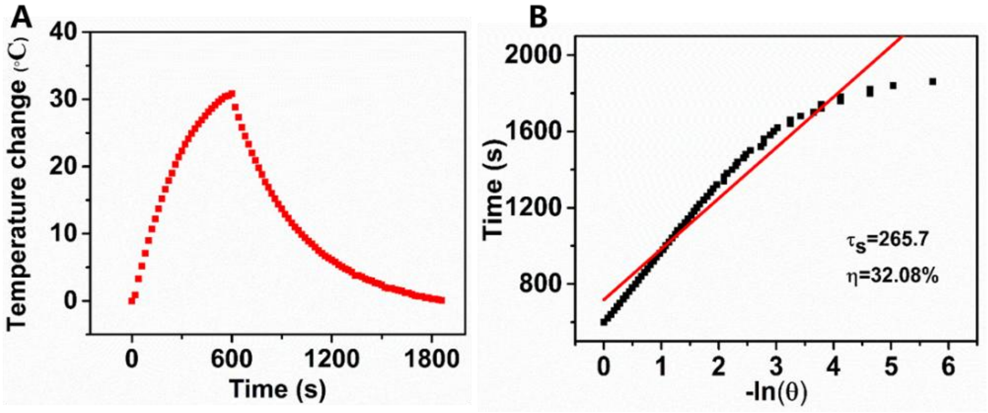

Fig. S10. Evaluation of photothermal conversion efficiency. (**A**) Photothermal effect of an aqueous solution of HA/PtP nanogels (Pt element, 50 μg/mL) under an 808 nm laser irradiation (0.8 W/cm^2). (**B**) The time constant for heat transfer was tested using the linear time data from the cooling period versus negative natural logarithm of driving force temperature obtained from the cooling stage.

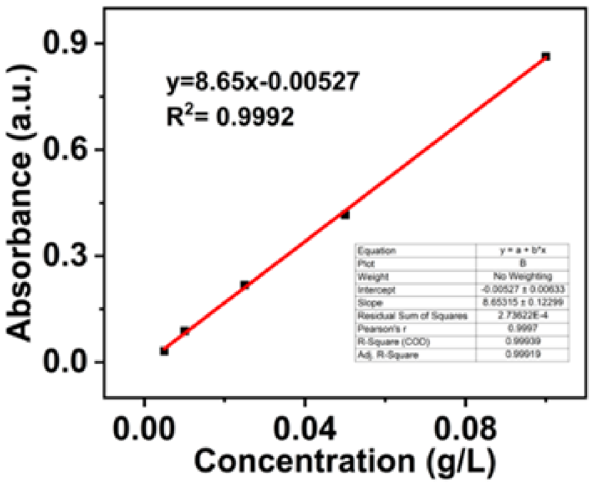

Fig. S11. Evaluation of extinction coefficient. The fitting curve of absorbance at 808 nm versus concentration for HA/PtP aqueous solutions.

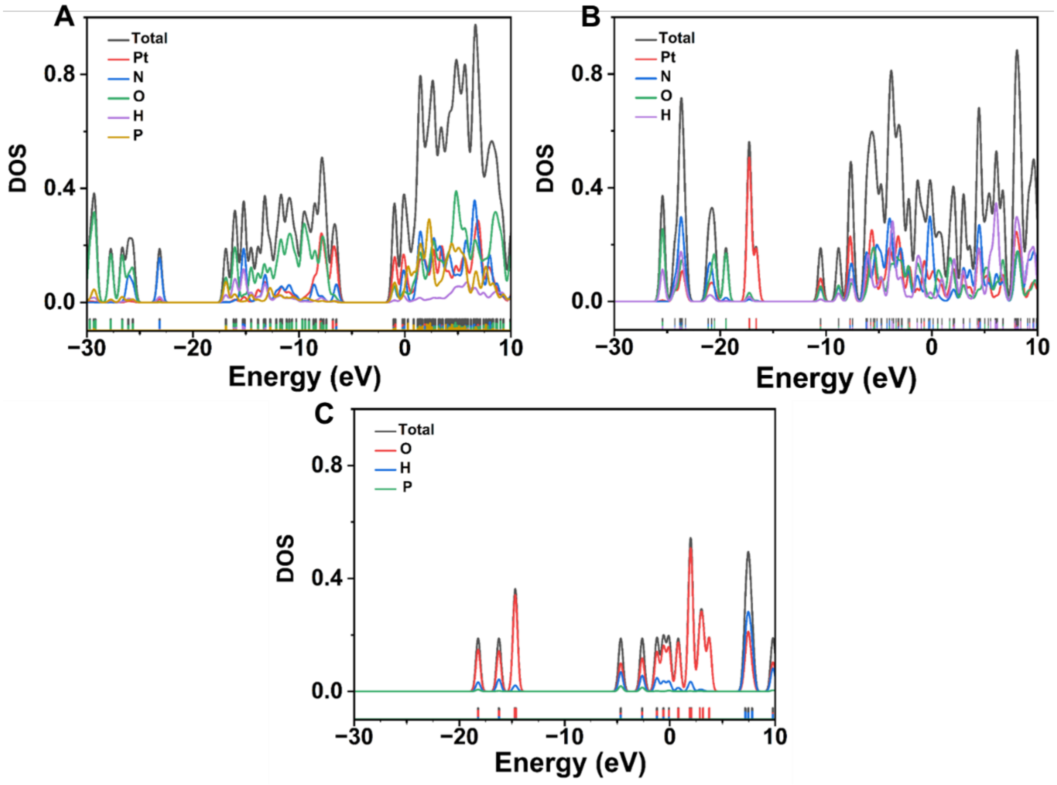

Fig. S12. Characterization of DOS for different components. DOS of (A) HA/PtP nanogels, (B) Cis and (C) $HPO4^{2-}$.

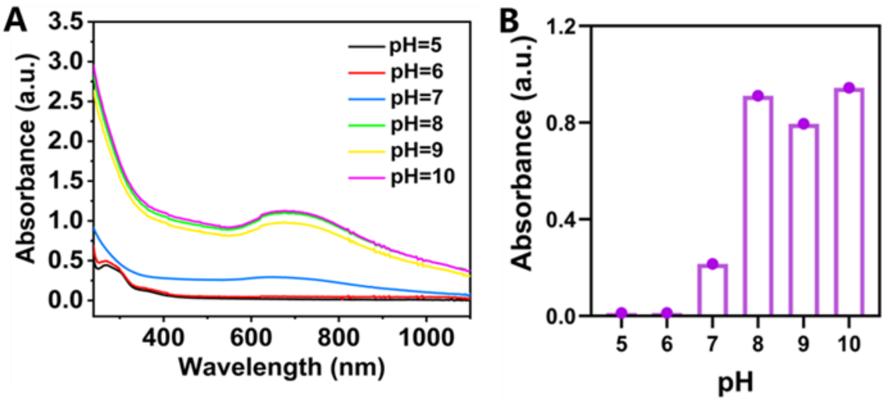

Fig. S13. Optimizing the synthesis conditions of the HA/PtP nanogels by varying pH. (**A**) The UV-vis absorption spectra of HA/PtP synthesized with PB buffer solution (0.1 mol/L) at different pH levels. (**B**) The corresponding absorbance at 808 nm.

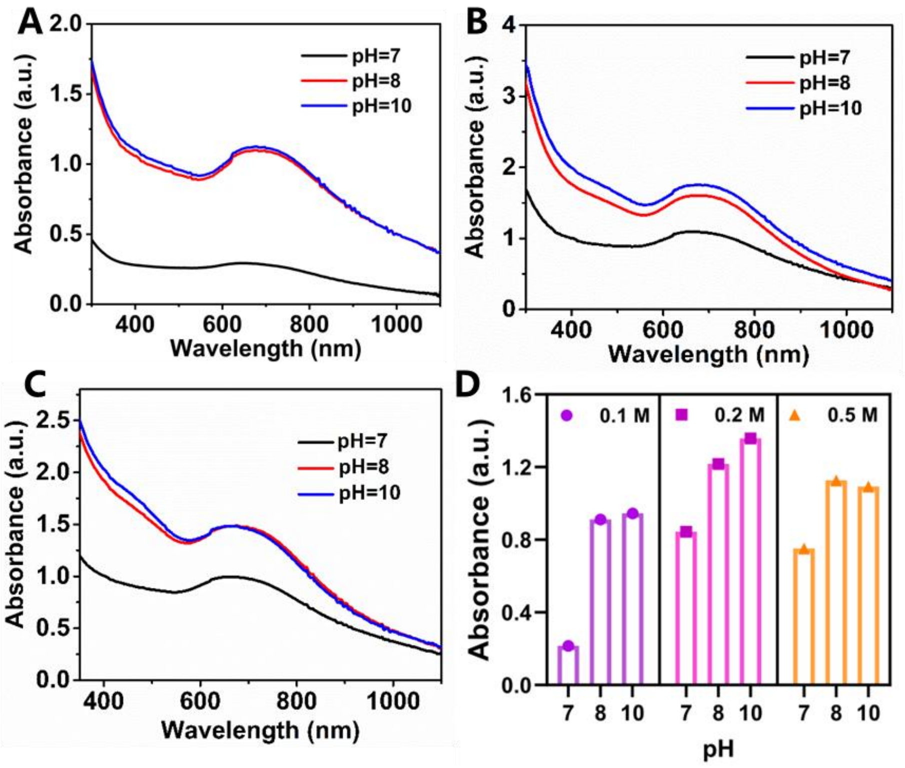

Fig. S14. Optimizing the synthesis conditions of the HA/PtP nanogels by varying concentration. (**A**-**C**) The UV-vis absorption spectra of HA/PtP synthesized with PB buffer solution at different concentrations (0.1, 0.2 and 0.5 mol/L). (**D**) The corresponding absorbance at 808 nm.

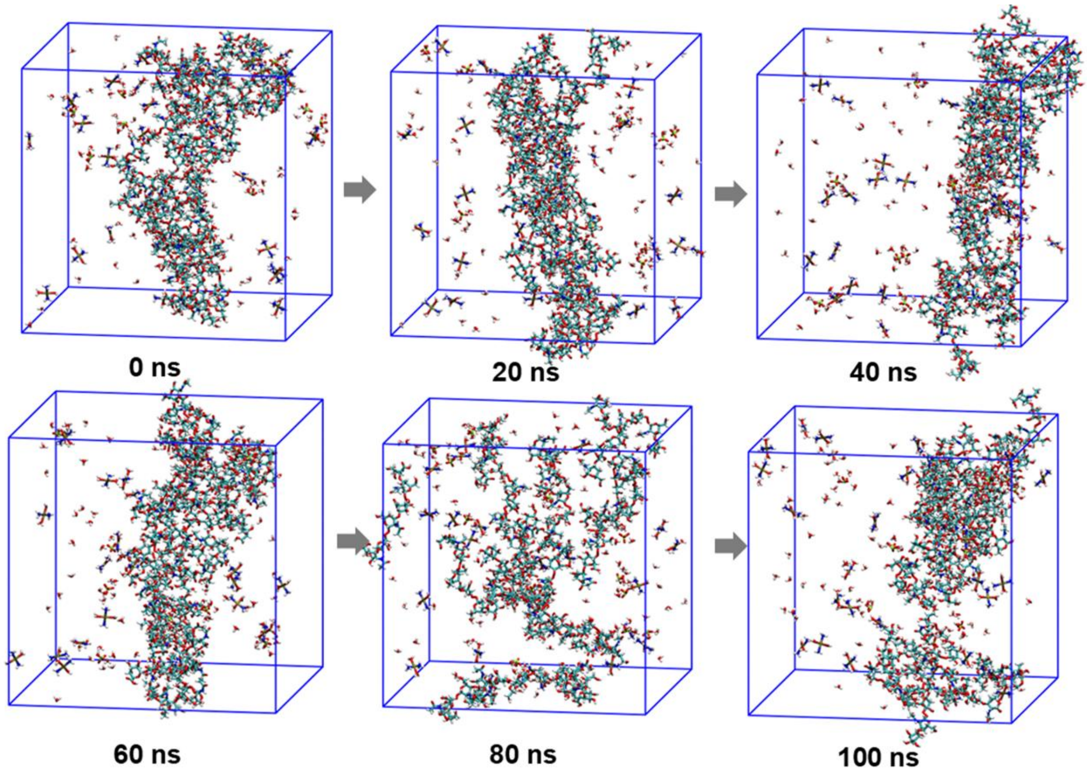

Fig. S15. MD simulation of the HA/PtP nanogels in an acidic condition. Time-dependent snapshots from MD simulation of nanogels formed by HA, Cis and $HPO4^{2-}$ in an acidic condition.

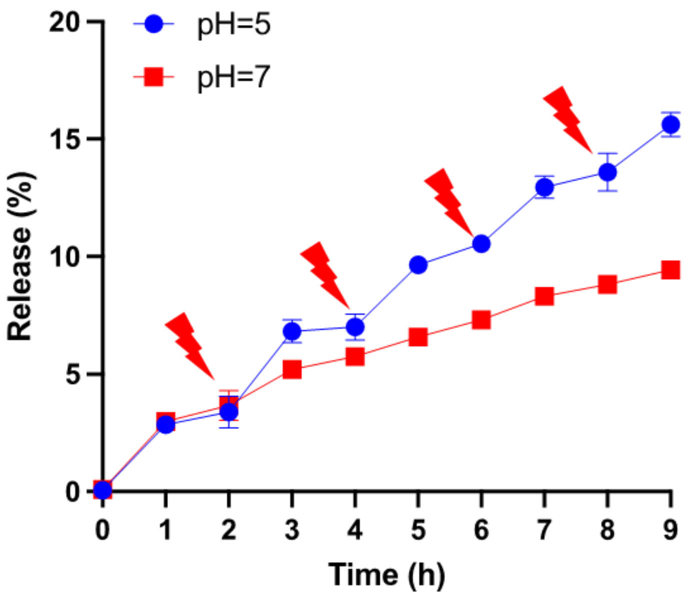

Fig. S16. Photothermally triggered release of Cis. Cis release ratio in PBS (pH =5, 7) irradiated with 0.8 W/cm^2 of an 808 nm laser for 10 min at 2, 4, 6 and 8 h.

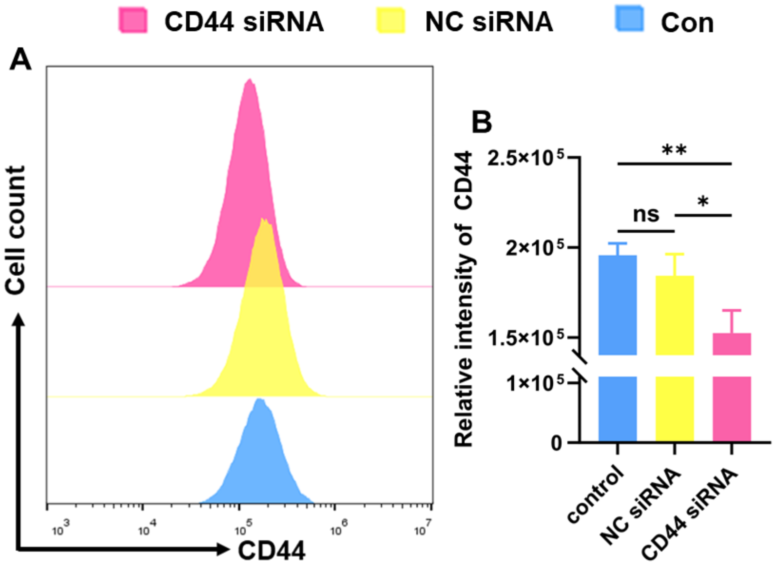

Fig.S17. Confirmation of CD44 knockdown. (**A**) Representative FACS graph of CD44 expression after the siRNA treatment. (**B**) Relative intensity of CD44 level. After the siRNA transfection, 4T1 cells were stained by APC-labeled CD44 antibody and analyzed by FACS (n=3).

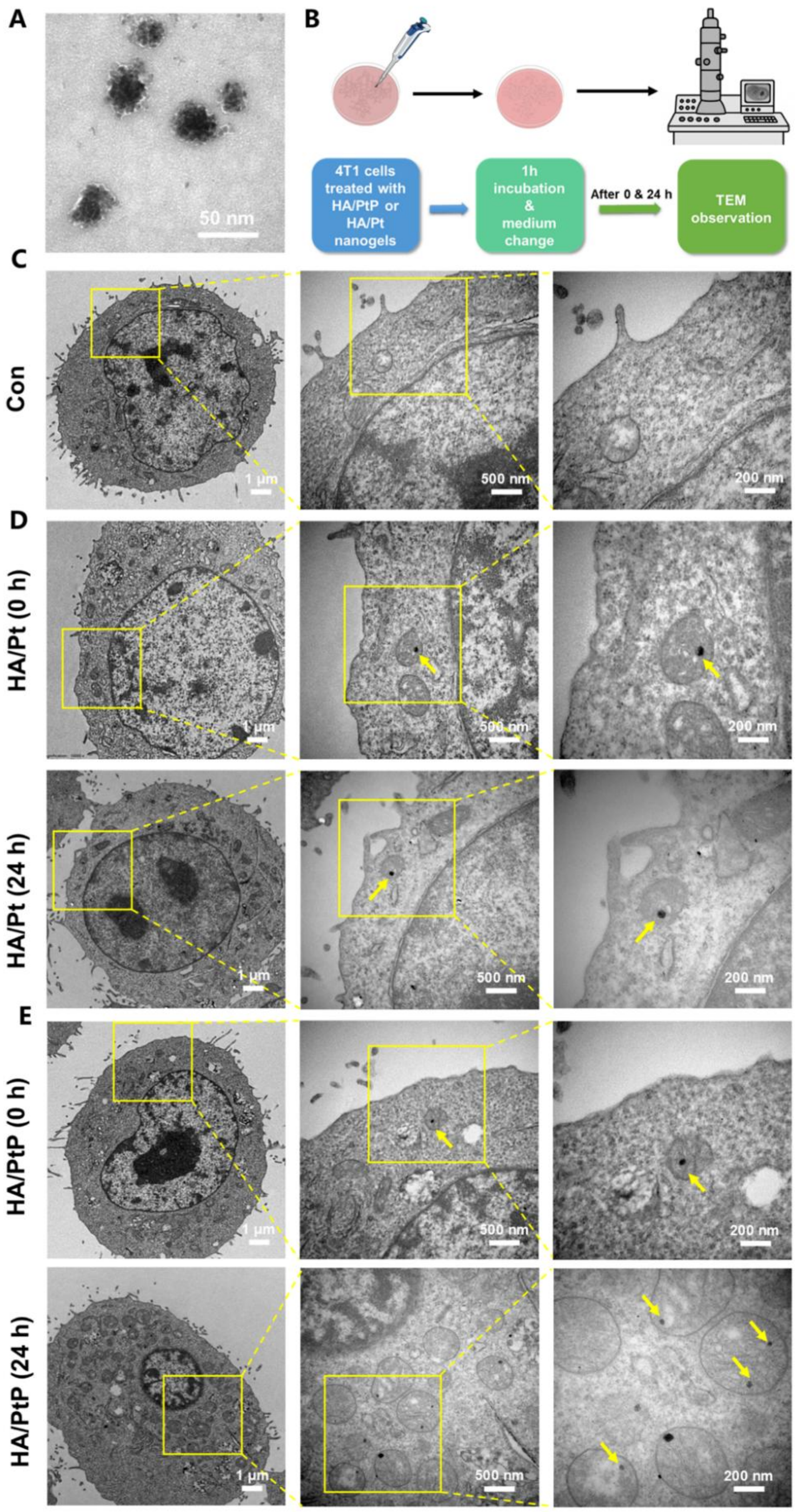

Fig. S18. Cellular TEM images of different nanogels. (**A**) TEM images of the HA/Pt nanogels with negative staining. (**B**) 4T1 Cells were pulsed by HA/Pt or HA/PtP nanogels for 1h and cultured in fresh medium, then TEM observations were performed after 0 and 24 hours of culture. (**C-E**) Representative TEM image of cells treated by medium only (C), HA/Pt (D) and HA/PtP (E).

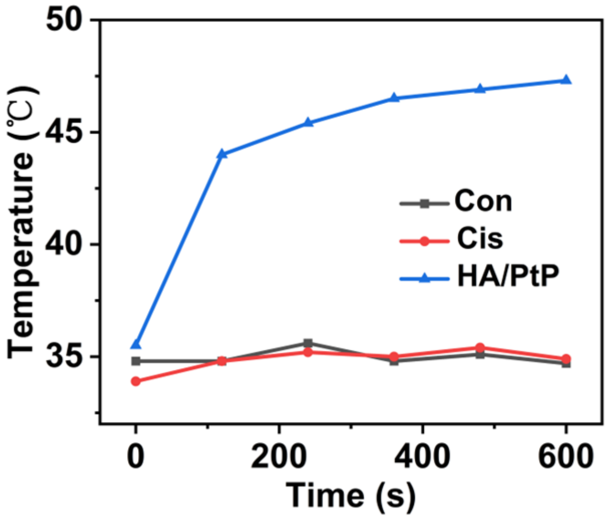

Fig. S19. *In vivo* temperature monitoring of tumors. Infrared thermography corresponding plot of temperature values of tumor-bearing mice treated with PBS, Cis and HA/PtP.

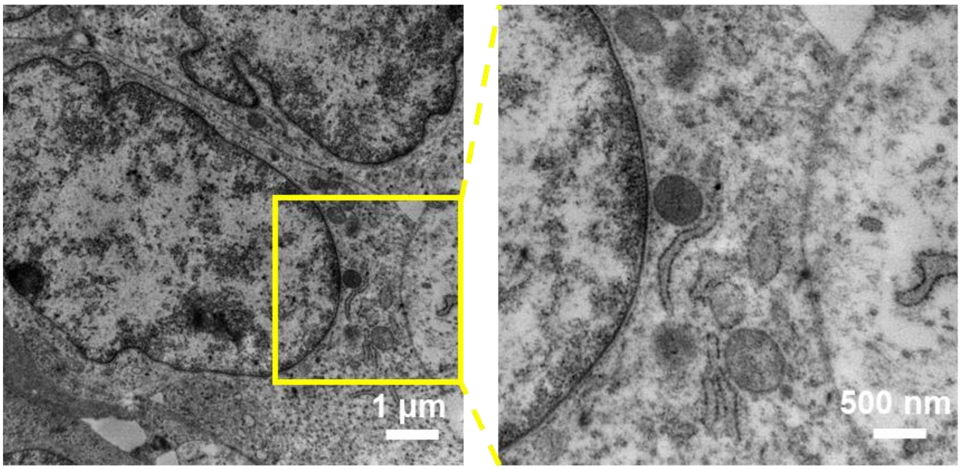

Fig. S20. Biological TEM imaging of control tumor. TEM image of 4T1 tumor after intravenous injection of saline for 72 h.

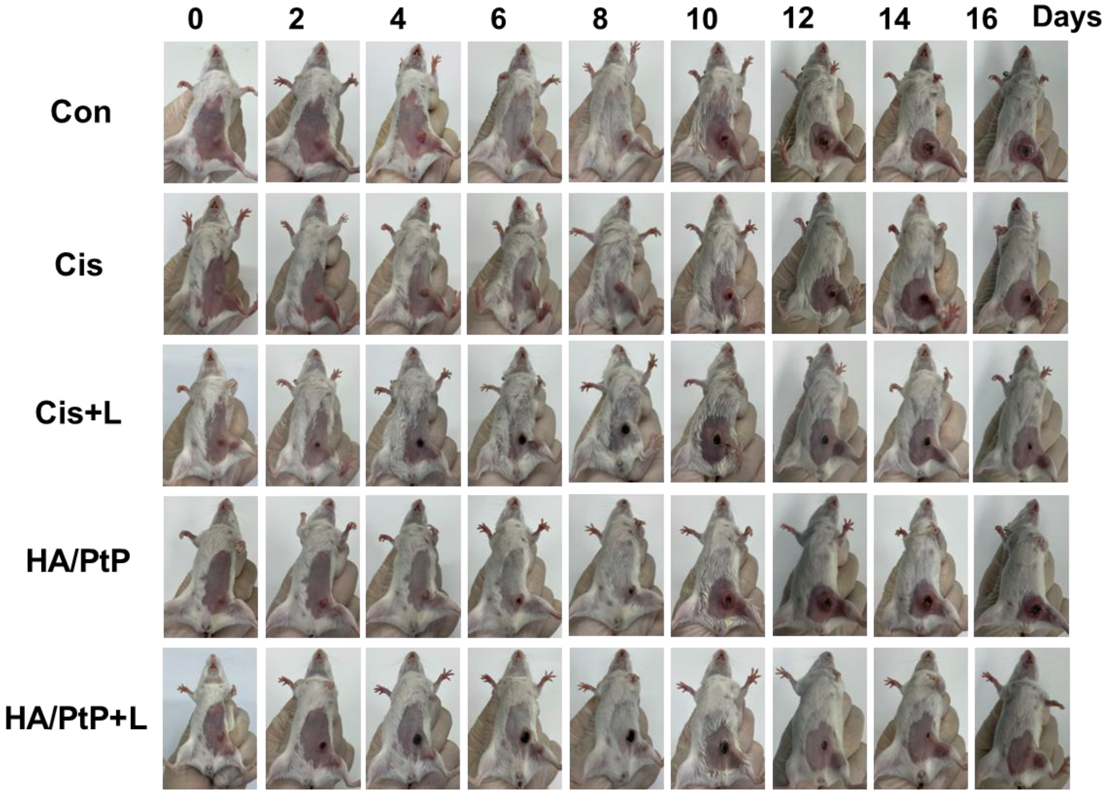

Fig. S21. Representative images of 4T1 tumor progression across treatment groups. Photographs of the 4T1 tumor-bearing mice at different time with different treatments.

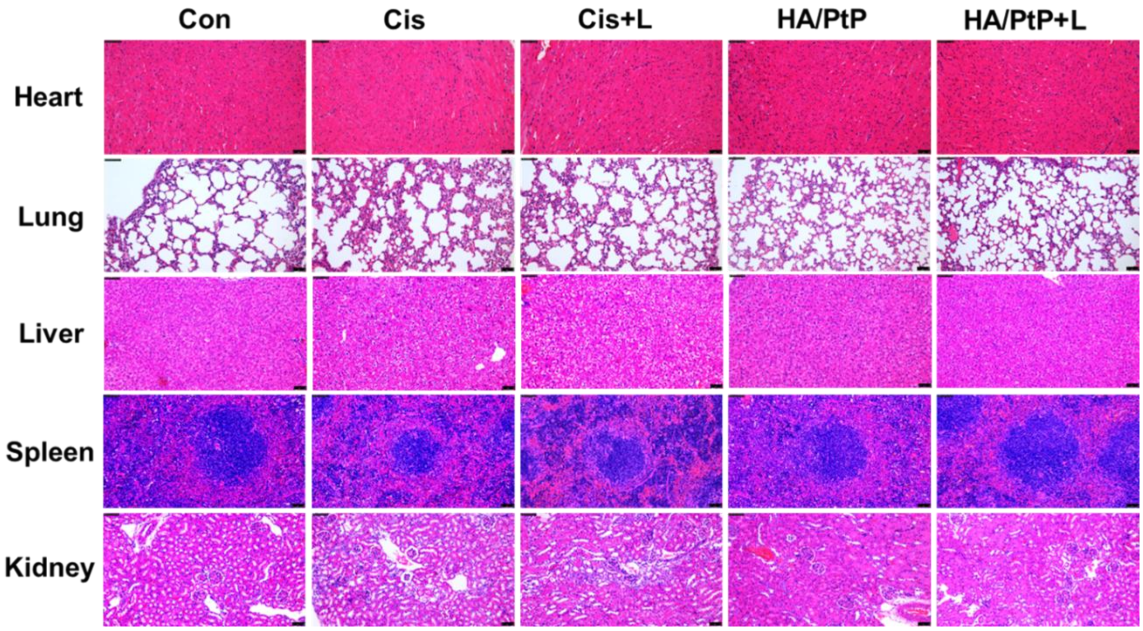

Fig. S22. Histopathological analysis of 4T1 tumor-bearing mice. H&E staining of tissue sections obtained from 4T1 tumor-bearing mice, including heart, liver, spleen, lung, and kidney with different treatments. Scale bar: 50 μm.

Tables

Table S1. Characterization of HA/Cis and HA/PtP dispersed in water (n=3).

	**HA/Cis**	**HA/PtP**
**Hydrodynamic size (nm)**	54.59±16.39	205.3±1.8
**PDI**	0.795±0.222	0.046±0.006
**Zeta potential (mV)**	-9.27±1.78	-38.00±0.21

Table.S2 LOI of HA, Cis, HA/Cis, Na_2HPO_4 and HA/PtP determined by TG analysis.

	**LOI**
Cis	33.89%
Na_2HPO_4	7.61%
HA	80.45%
HA/Cis	82.15%
HA/PtP	66.54%

Table.S3 The hydrodynamic size and PDI of HA/PtP nanogels prepared based on the PBS buffer of different pH and concentrations (n=3). PB buffer (pH 10, 0.2 M) was selected to prepare HA/PtP in this study.

PB buffer pH	PB buffer concentration	Z-average (d.nm)	PDI
pH=10	0.1 M	241.8±3.97	0.380±0.021
	0.2 M	194.7±1.22	0.072±0.020
	0.5 M	307.9±4.18	0.108±0.022
pH=8	0.1 M	261.0±4.26	0.413±0.011
	0.2 M	321.8±6.59	0.157±0.052
	0.5 M	350.8±4.50	0.074±0.014
pH=7	0.1 M	314.6±3.73	0.154±0.021
	0.2 M	342.2±7.77	0.107±0.064
	0.5 M	608.8±29.49	0.528±0.114
pH=6	0.1 M	596.6±25.60	0.714±0.390
pH=5	0.1 M	1037.0±311.40	1.000±0.008

Table S4. The number of hydrogen bonds formed in alkaline systems. MD simulations performed with OH^- input, corresponding to Fig. 2B.

	**Polymer**	**Pt**	**P**	**OH^-**	**H_2O**
**Polymer**	107.1	-	-	-	-
**Pt**	1.2	0.08	-	-	-
**P**	0.1	13.7	0	-	-
**OH-**	9.0	87.1	0.2	0.2	-
**H_2O**	515.2	115.6	52.8	499.1	17217.2

Table S5. The number of hydrogen bonds formed in acidic systems. MD simulation performed with H^+ input, corresponding to fig S12.

	**Polymer**	**Pt**	**P**	**H_3O^+**	**H_2O**
**Polymer**	106.9	-	-	-	-
**Pt**	2.3	0.05	-	-	-
**P**	0.6	8.8	0	-	-
**H_3O^+**	1.6	0.08	91.6	0.002	-
**H_2O**	511.8	193.8	29.3	208	17002.9

Table S6. The composition of each simulation system.

	Polymer	Pt	P	H_3O^+/OH^-	H_2O
**Alkaline system**	20	24	12	100	10000
**Acidic system**	20	24	12	100	10000

Movies

Movie S1. The MD simulation video illustrates the dynamic behavior of OH^- input, as described in Fig. 2B.

Movie S2. The MD simulation video illustrates the dynamic behavior of H^+ input, as described in fig. S12.
